# Influence of Polarization Temperature and Time on the Electromechanical Performance of Commercial PZT-4 Ceramics

**DOI:** 10.3390/ma19122656

**Published:** 2026-06-20

**Authors:** Bruna Karina da Silva Oliveira, Douglas Santos Silva, Raí Felipe Pereira Junio, João Gabriel Passos Rodrigues, Rubens Lincoln Santana Blazutti Marçal, Sergio Neves Monteiro, Priscila Simões Teixeira Amaral, Roberto da Costa Lima, Foluke Salgado de Assis

**Affiliations:** 1 Chemistry and Materials Technology Group, Brazilian Navy Research Institute–IPqM, Rua Ipiru, 02, Cacuia, Ilha do Governador, Rio de Janeiro CEP 21931-095, RJ, Brazil; brunaoliveira@eq.ufrj.br (B.K.d.S.O.); raivsjfelipe@ime.eb.br (R.F.P.J.); jgprodrigues@ima.ufrj.br (J.G.P.R.); priscila.simoes@marinha.mil.br (P.S.T.A.); costalima.roberto@marinha.mil.br (R.d.C.L.); foluke.assis@marinha.mil.br (F.S.d.A.); 2 Department of Chemical Engineering, Federal University of Rio de Janeiro–UFRJ, Av. Athos da Silveira Ramos, 149, Cidade Universitária, Rio de Janeiro CEP 21941-909, RJ, Brazil; 3 Department of Materials Science, Military Institute of Engineering–IME, Praça General Tiburcio, 80, Praia Vermelha, Urca, Rio de Janeiro CEP 22290-270, RJ, Brazil; sergio.neves@ime.eb.br; 4 Department of Materials Engineering, National Service for Industrial Training–SENAI, Parque Tecnológico da UFRJ, Rua Leopoldo de Meis, nº 301, Cidade Universitária, Rio de Janeiro CEP 21941-855, RJ, Brazil; rubenslsbm@gmail.com; 5 Local Development, Augusto Motta University Center—UNISUAM, Avenida Paris, 84, Bonsucesso, Rio de Janeiro CEP 21041-020, RJ, Brazil

**Keywords:** piezoelectric ceramics, polarization optimization, electromechanical properties, Avrami model, statistical analysis

## Abstract

Commercial lead zirconate titanate (PZT) ceramics are widely employed in electromechanical devices due to their excellent piezoelectric response and operational stability. This study investigates the influence of polarization temperature and time on the electromechanical performance of commercial Sparkler PZT-4 (Navy Type I) ceramics. Samples were compacted, sintered at 1230 °C, and polarized under temperatures ranging from 80 to 110 °C for 2, 8, and 15 min using a constant electric field of 3.0 kV/mm. Microstructural, physical, and crystallographic analyses confirmed the successful processing of the ceramics, yielding an apparent density of 7.68 g/cm^3^, relative density of 96.02%, and the predominance of the tetragonal Pb(Zr,Ti)O_3_ perovskite phase. Electromechanical characterization revealed a strong dependence of the piezoelectric coefficient (d_33_) and electromechanical coupling factor (K_p_) on the polarization conditions. Maximum values of d_33_ = 325.8 pC/N and K_p_ = 0.509 were obtained under elevated temperatures and longer polarization times. A phenomenological Avrami approach indicated faster apparent domain alignment at higher temperatures, while ANOVA and Tukey tests confirmed the significant influence of polarization parameters on the electromechanical response. The results identify favorable polarization conditions for commercial PZT-4 ceramics used in sensors, actuators, and ultrasonic transducers.

## 1. Introduction

Piezoelectric ceramics based on lead zirconate titanate (PZT) remain among the most important functional materials used in modern electromechanical devices due to their excellent piezoelectric response, strong electromechanical coupling, and relatively stable dielectric behavior over a broad range of operating conditions [1,2,3,4,5]. These characteristics make PZT ceramics essential in a wide variety of technological applications, including ultrasonic transducers, sonar systems, sensors, actuators, vibration control systems, and energy harvesting devices [6]. Owing to their high electromechanical conversion efficiency and long-term operational stability, PZT-based materials continue to represent the benchmark for many high-performance piezoelectric systems [6,7,8,9].

The electromechanical behavior of PZT ceramics is strongly governed by the complex interplay between processing parameters, microstructural evolution, crystallographic structure, and polarization efficiency [6]. Microstructural characteristics such as particle morphology, agglomeration state, packing during compaction, and grain growth during sintering can significantly influence densification behavior and domain mobility [10]. These factors ultimately affect dielectric properties, piezoelectric response, and electromechanical coupling efficiency. In addition, the crystallographic stability of the perovskite phase and the possible presence of secondary phases play an important role in determining the functional performance of the material [10,11].

The polarization process represents a critical step in the activation of the piezoelectric properties of ferroelectric ceramics. During polarization, the application of a strong electric field promotes the alignment of ferroelectric domains, generating a remanent polarization responsible for the electromechanical response of the material [12,13]. However, the efficiency of this process is strongly dependent on several parameters, including electric field intensity, polarization temperature, and polarization time [6,14,15]. These variables influence domain wall mobility, polarization kinetics, and stabilization of the ferroelectric state, thereby directly affecting the resulting values of the longitudinal piezoelectric coefficient (d_33_) and the electromechanical coupling factor (K_p_).

Although PZT ceramics have been extensively studied over the past decades, most investigations have focused on either compositional modification, dopant effects, or individual aspects of processing and functional characterization [16]. In many cases, microstructural characterization, crystallographic analysis, densification behavior, and electromechanical performance are investigated separately [17]. As a result, integrated studies that simultaneously correlate powder morphology, microstructural evolution during sintering, crystallographic stability, and polarization optimization remain relatively limited, particularly when commercially available PZT powders are considered.

Commercial PZT powders often exhibit complex microstructural characteristics, including particle agglomeration, broad particle size distributions, and variations in chemical homogeneity depending on synthesis and processing routes [6]. These features can influence powder packing during compaction, densification behavior during sintering and the efficiency of domain alignment during polarization [6,17,18]. Consequently, a comprehensive investigation integrating microstructural, structural, physical, and electromechanical analyses is required to establish reliable processing–structure–property relationships for these materials.

Furthermore, while polarization conditions are known to significantly affect the electromechanical performance of PZT ceramics, systematic studies aimed at statistically evaluating the combined influence of polarization parameters, such as electric field, temperature, and time are still scarce in the literature. In particular, the use of statistical tools to define optimized polarization windows capable of maximizing the functional response of commercial PZT-4 ceramics remains relatively unexplored. Such an approach can provide important insights into polarization kinetics and contribute to the development of more reliable and efficient electromechanical devices.

In this context, the present work aims to investigate the processing–structure–property relationships in commercial Sparkler PZT-4 (Navy Type I) ceramics through a comprehensive experimental approach. The study integrates scanning electron microscopy (SEM), energy-dispersive spectroscopy (EDS), density and water absorption measurements, X-ray diffraction with Rietveld refinement, and electromechanical characterization. Special emphasis is placed on correlating powder morphology, densification behavior, crystallographic stability, and polarization parameters with the resulting functional properties of the material.

In addition, a statistically supported analysis of polarization conditions is performed using ANOVA and Tukey tests in order to identify optimized combinations of polarization temperature and polarization time under a fixed electric-field condition of 3.0 kV/mm capable of maximizing the electromechanical response. Special emphasis is placed on correlating powder morphology, densification behavior, crystallographic stability, and polarization parameters with the resulting functional properties of the material. By establishing consistent processing–structure–property relationships, this work provides practical guidelines for optimizing polarization conditions and manufacturing procedures for commercial PZT-4 ceramics used in sensors, actuators, ultrasonic transducers, and other electromechanical devices.

## 2. Materials and Methods

### 2.1. Raw Material and Sample Preparation

Commercial lead zirconate titanate powder classified as PZT-4 (Navy Type I) and supplied by Sparkler Ceramics (Pune, India) was used as the starting material. According to the manufacturer, the material corresponds to the perovskite structure Pb(Zr,Ti)O_3_, typically employed in high-power piezoelectric devices such as ultrasonic transducers due to its high mechanical quality factor and stable dielectric response. The powder was used as received, without additional chemical modification.

Prior to processing, the material was gently homogenized to reduce possible agglomeration effects resulting from storage and handling. Green bodies were prepared by uniaxial compaction using a hardened steel die to produce cylindrical pellets suitable for structural and electromechanical characterization.

The compacted samples were subsequently sintered in an electric furnace under an air atmosphere. The thermal cycle started at room temperature with a preheating stage at 75 °C for 5 min, followed by a temperature stabilization step at 200 °C for an additional 5 min. The specimens were then heated to the sintering temperature of 1230 °C at a rate of 12 °C/min and held at this temperature for 200 min to promote densification and microstructural development. After sintering, the samples were cooled under controlled conditions to 150 °C at a cooling rate of 15 °C/min and subsequently allowed to cool naturally to room temperature. These processing parameters were selected to ensure adequate densification, promote grain growth and interparticle bonding, minimize residual porosity, and preserve the crystallographic stability of the PZT ceramic phase.

#### Samples Groups

Table 1 summarizes the grouping of the sintered PZT samples according to the polarization conditions adopted in this study, including polarization time, temperature, and applied electric field. The sample nomenclature reflects these parameters, allowing a clear identification of each experimental condition (time/temperature/electric field). The electric field was maintained constant at 3.0 kV/mm throughout the study in order to isolate the influence of polarization temperature and polarization time on the electromechanical response of the ceramics. This field level was selected based on manufacturer recommendations and values commonly reported for commercial PZT-4 ceramics, being sufficiently high to promote effective ferroelectric domain alignment while minimizing the risk of dielectric breakdown or electrical degradation. Therefore, the present experimental design was focused on evaluating the combined effects of temperature and time under a fixed electric-field condition.

The variation in polarization time and temperature was designed to systematically investigate their influence on domain alignment and, consequently, on the electromechanical properties of the PZT samples. The electric field was maintained constant at 3.0 kV/mm throughout the study in order to isolate the effects of polarization temperature and polarization time on the electromechanical response. This field level was selected based on manufacturer recommendations and values commonly reported for commercial PZT-4 ceramics, being sufficiently high to promote effective ferroelectric domain alignment while minimizing the risk of dielectric breakdown or electrical degradation. Thus, the polarization time (2, 8, and 15 min) and temperature (80, 90, 100, and 110 °C) were varied to promote different levels of domain reorientation under a fixed electric-field condition. These parameters directly affect the apparent evolution of domain switching and polarization efficiency, making them suitable inputs for the phenomenological application of the Avrami model. By correlating the experimentally obtained d_33_ values with the polarization conditions, it was possible to construct Avrami-type curves and evaluate the apparent fraction of aligned domains as a function of time and temperature. This approach provides a comparative description of the polarization evolution under different processing conditions and allows the identification of polarization conditions that maximize the functional response of the material.

### 2.2. Microstructural Characterization

The morphology and microstructural characteristics of the PZT powder were analyzed by scanning electron microscopy (SEM) using a QUANTA 250 FEG microscope equipped with a field emission gun (FEG), located at the Electron Microscopy Laboratory of the Military Institute of Engineering (LME-IME). Powder samples were mounted on aluminum stubs using conductive carbon tape. SEM images were acquired using secondary electron detection with an acceleration voltage of 20 kV, beam aperture of 5, working distance ranging from 9.8 to 17.2 mm, and spot size of 5. Micrographs were obtained at different magnifications in order to evaluate particle morphology, agglomeration state, and surface features. The agglomerate size was determined from images acquired at 500× magnification using digital image analysis.

Energy-dispersive X-ray spectroscopy (EDS) coupled to the SEM system investigated the chemical composition. Analyses were performed at an acceleration voltage of 20 kV using ESPRIT software 2.1 (Bruker Nano GmbH, Berlin, Germany) for spectral acquisition and processing. Quantitative evaluation was conducted using area scan mode to reduce the influence of local heterogeneities associated with individual particles. In addition, elemental mapping was performed to assess the spatial distribution of the main elements present in the PZT structure and to verify the compositional homogeneity of the analyzed regions.

### 2.3. Structural Characterization

The crystalline structure of the samples was analyzed by X-ray diffraction (XRD) using an X’PERT PRO MRD diffractometer (PANalytical, Almelo, The Netherlands). Measurements were performed using Co-Kα radiation (λ = 1.789 Å) operating at 40 kV and 30 mA. Diffraction patterns were collected in the 2θ range from 20° to 80° with a step size of 0.02°. Phase analysis was carried out using HighScore Plus software (version 4.9, Malvern Panalytical, Almelo, The Netherlands), while structural refinement was performed using the Rietveld method implemented in FullProf Suite (version 7.80, Institut Laue-Langevin, Grenoble, France). This procedure allowed the identification of the crystalline phases present in the samples and the evaluation of the crystallographic parameters associated with the perovskite structure.

### 2.4. Physical Characterization

The density of the sintered samples was determined using the Archimedes method. The procedure involved measuring the dry mass of the specimen in air, the saturated mass after immersion in water, and the submerged mass. From these values, the apparent density, water absorption, and apparent porosity were calculated according to Archimedes’ principle. Multiple specimens were analyzed to ensure reproducibility, and the results were expressed as mean values accompanied by their respective standard deviations. The relative density was estimated by comparing the experimental density with the theoretical density of PZT ceramics.

### 2.5. Electromechanical Characterization

To activate the piezoelectric response of the ceramics, the sintered samples were subjected to a polarization process. Prior to polarization, both faces of each specimen were coated with silver electrodes to enable the application of the electric field. The metallized samples were subsequently heat treated at 590 °C for 1 h to ensure adequate adhesion and electrical conductivity of the electrodes. Polarization was then carried out by applying a high DC electric field across the thickness of the samples according to the conditions described in Table 1.

After polarization, the longitudinal piezoelectric coefficient (d_33_) was measured using the Berlincourt quasi-static method with a C-HEMI YE2730A d_33_ meter (ATCP Engenharia Física, São Carlos, SP, Brazil). The load coefficient (d_33_) can be measured by applying a controlled, predefined force to the thickness of the specimen in the same direction as the polarization. The experimental values obtained for the samples under study were compared to the PZT standard (ATCP Engenharia Física) with a d_33max_ value of 330 pC/N.

The electromechanical response of the polarized samples was further evaluated using a TRZ^®^ G10 transducer and sonotrode analyzer (ATCP Engenharia Física, São Carlos, SP, Brazi) operating with TRZ^®^ 7.0 software in a frequency range up to 200 kHz. The planar coupling factor (K_p_) is calculated from the relationship expressed in Equation (1), using the resonance and anti-resonance frequency values of the specimens measured by the TRZ analyzer.

(1) $$K_{p}=\sqrt{\left(\frac{2.51(f_{a}-f_{r})}{f_{a}}\right)-{\left(\frac{f_{a}-f_{r}}{f_{a}}\right)}^{2}}$$

Electrical parameters associated with the equivalent circuit of the samples were also obtained using a Minipa MC-155 LCR meter (Minipa do Brasil Ltd., São Paulo, SP, Brazil), enabling the correlation between dielectric properties and piezoelectric performance.

### 2.6. Application of Avrami Model

The Johnson–Mehl–Avrami–Kolmogorov (JMAK) formalism [19,20,21,22,23] was employed in the present study as a phenomenological approach to comparatively describe the evolution of ferroelectric domain alignment during the polarization process. Although originally developed to represent phase transformation kinetics involving nucleation and growth mechanisms [24,25,26], the Avrami model has also been used as a simplified kinetic framework to describe saturation-type evolution in functional materials systems. In the present work, the model is applied exclusively as a comparative tool to evaluate the influence of polarization time and temperature on the apparent evolution of domain alignment in the 33 direction. The general form of the Avrami equation is presented in Equation (2):

(2) $$Y\left(t\right)=1-exp(-kt^{n})$$

where Y(t) represents the normalized fraction associated with the apparent evolution of domain alignment at time t, k is the apparent temperature-dependent kinetic constant, and n is the apparent Avrami exponent associated with the overall kinetic profile of the polarization process. In the present study, these parameters are interpreted only from a phenomenological standpoint, without direct attribution to classical nucleation-and-growth mechanisms. The model is therefore employed as a comparative kinetic approximation for evaluating the influence of polarization conditions on the evolution of the electromechanical response.

In the context of this study, the transformed fraction was associated with the fraction of ferroelectric domains aligned in the 33 direction, being estimated from the ratio between the experimental piezoelectric coefficient and the theoretical maximum value of the material in Equation (3):

(3) $$Y\left(t\right)=\frac{d_{33}(t)}{d_{33,max}}$$

where d_33_(t) corresponds to the experimentally measured value after the polarization process, and d_33,Max_ = 330 pC/N represents the assumed saturation value for PZT. This normalization allows the piezoelectric coefficient to be interpreted as an indirect measure of the fraction of effectively aligned domains.

The temperature dependence of kinetic parameters is commonly described in the literature using Arrhenius-type relationships. In the present work, this formalism is mentioned only as a theoretical reference to illustrate the expected influence of temperature on the polarization kinetics. However, no complete Arrhenius fitting or activation energy determination was performed, since the experimental dataset was limited to comparative kinetic evaluation under the investigated polarization conditions.

The kinetic parameters k and n were also determined through the linearization of the Avrami equation, enabling a more reliable extraction of the fitting constants from the experimental data. By applying the double-logarithmic transformation of the original model reported in Equation (4):

(4) $$\ln\left[-\ln\left(1-Y\left(t\right)\right)\right]=\ln k+n\;\ln t$$

A linear relationship is obtained between lnt and $\ln\left[-\ln\left(1-Y\left(t\right)\right)\right]$ in which the slope corresponds to the Avrami exponent n and the intercept is equal to ln k. This procedure allows a straightforward kinetic interpretation of the polarization process and provides a consistent method to compare the effects of temperature on the domain alignment behavior of the PZT samples.

Thus, the Avrami model was applied considering polarization time and temperature as control variables, while the d_33_ parameter was used as a macroscopic indicator of the evolution of domain alignment. This approach allows the polarization process to be comparatively analyzed as a time-dependent domain-alignment phenomenon. The analysis of the fitting parameters k and n obtained from the Avrami equation was used only to comparatively evaluate the kinetic evolution of the polarization process under different thermal conditions. In this context, the parameters should not be interpreted in terms of classical nucleation-and-growth mechanisms, but rather as phenomenological indicators associated with the rate of domain alignment and the tendency toward polarization saturation.

It is important to emphasize that the use of the Avrami formalism in the present study does not imply that the polarization process corresponds to a true nucleation-and-growth phase transformation in the classical thermodynamic sense. Instead, the model is employed exclusively as a phenomenological and comparative kinetic framework to describe the temporal evolution of ferroelectric domain alignment under different polarization conditions. Therefore, the transformed fraction Y(t), estimated from normalized d_33_ values, should be interpreted only as an indirect macroscopic indicator of polarization efficiency rather than a direct measurement of a physically transformed phase fraction.

For clarity, the terminology adopted in this work follows the mathematical formulation traditionally associated with the Avrami equation. Terms such as “apparent transformed fraction”, “apparent kinetic constant”, and “apparent Avrami exponent” are used exclusively as fitting descriptors derived from the phenomenological model and should not be interpreted as direct representations of physical nucleation rates, growth mechanisms, or actual transformed phase fractions in the ferroelectric ceramic.

### 2.7. Statistical Analysis

Statistical analysis was performed to evaluate the effects of polarization temperature and polarization time on the electromechanical response of the sintered PZT-4 ceramics. A two-factor experimental design was adopted, considering polarization temperature as the first factor, with four levels (80, 90, 100, and 110 °C), and polarization time as the second factor, with three levels (2, 8, and 15 min). The electric field was kept constant at 3.0 kV/mm for all experimental groups in order to isolate the effects of temperature and time.

The response variables analyzed were capacitance (C_p_), longitudinal piezoelectric coefficient (d_33_), and planar electromechanical coupling factor (K_p_). The results were expressed as mean values ± standard deviation. The influence of polarization temperature, polarization time, and their interaction was evaluated using two-way analysis of variance (two-way ANOVA). Statistical significance was considered at *p* ≤ 0.05. When significant differences were detected, Tukey’s post hoc test was applied to identify statistically different groups.

In addition to *p*-values, effect size parameters were calculated to quantify the relative contribution of each factor to the total variability of the response variables. Eta-squared (η^2^), partial eta-squared (η^2^p), omega-squared (ω^2^), and partial omega-squared (ω^2^p) were used to assess the magnitude of the effects associated with temperature, time, and their interaction. The significance levels were reported as *p* ≤ 0.05, *p* ≤ 0.01, and *p* ≤ 0.001.

## 3. Results and Discussion

### 3.1. Powder Characterization

**Figure 1:**
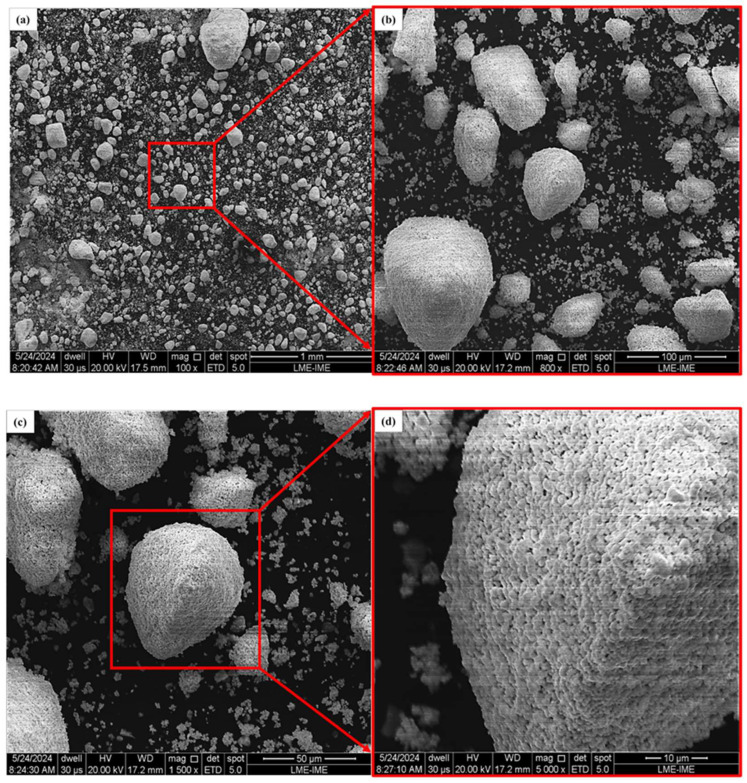
SEM micrographs of PZT-4 Sparkler powder at different magnifications: (**a**) 100×, showing polydisperse distribution of particles and agglomerates; (**b**) 800×, highlighting subspheroidal granules with a rough surface; (**c**) 1500×, showing hierarchical agglomerated structure; (**d**) 5000×, revealing micrometric subunits and interparticle porosity.

The morphology of PZT-4 Sparkler powder is shown in Figure 1, which illustrates the polydisperse particle distribution and the formation of hierarchical agglomerates at different magnification levels.

Scanning electron microscopy (SEM) micrographs of PZT-Sparkler samples reveal a heterogeneous and strongly polydisperse morphology, characterized by the coexistence of fine particles and larger granules. At lower magnification at 100× (Figure 1a), a wide size distribution is observed, in which micrometric and submicrometric particles are dispersed among agglomerates and granules with dimensions reaching hundreds of micrometers. This behavior indicates that the material is not predominantly composed of isolated primary particles, but rather a hierarchical agglomeration system, in which fine fractions coexist with larger granules, partially filling the intergranular voids. This characteristic may favor powder packing during shaping, although it can also introduce local density heterogeneities in the green body.

With increased magnification at 800× (Figure 1b), the granules are found to have a rounded to sub-spheroidal morphology, with smoothed contours and an absence of well-defined facets, suggesting that they are not individual crystalline particles, but aggregates formed during previous processing steps. The surface of these granules is markedly rough, with an irregular appearance, evidencing the presence of smaller adhered particles and secondary agglomerates distributed around and on their surface.

In Figure 1c intermediate magnifications (1500×), it becomes clearer that the granules are structurally porous, consisting of strongly agglomerated micrometric subunits. The presence of free fine particles surrounding these granules reinforces the multimodal nature of the particle size distribution, suggesting that, during shaping, these agglomerates may partially or totally fragment, modifying the effective size distribution and influencing the homogeneity of the packing.

At higher magnification (5000×), detailed analysis of the granule surface reveals the presence of primary particles or sub-agglomerates with dimensions on the order of a few micrometers, separated by micro voids and open pores. Maintaining this porosity after densification can negatively impact the electrical and piezoelectric properties of the material, since pores act as dielectric discontinuities, reducing the effective dielectric constant and hindering the homogeneous polarization of PZT.

**Figure 2:**
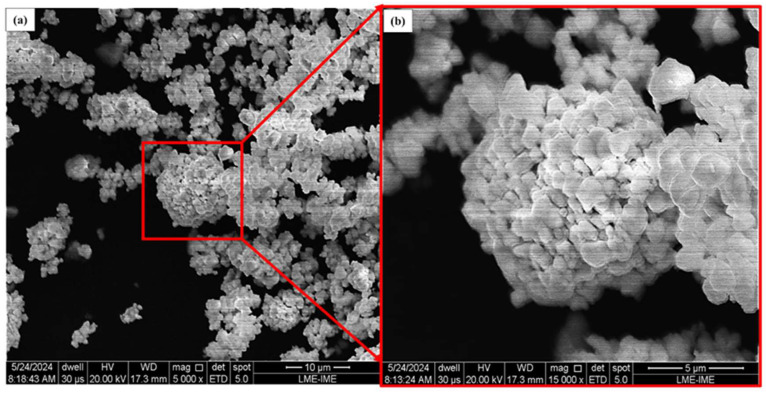
SEM micrographs of PZT-4 Sparkler powder: (**a**) 5000×, showing agglomerates composed of micrometric primary particles; (**b**) 15,000×, detail of the highlighted region, showing faceted/polygonal particles and interparticle porosity.

The morphology of the clusters at higher magnification is shown in Figure 2, highlighting the composition of the granules by faceted primary particles and the presence of interparticle porosity, characteristic of a poorly coalesced cluster structure.

SEM micrographs of PZT-Sparkler at magnifications of 5000× (a) and 15,000× (b) consistently show that the material is predominantly in the form of clusters composed of smaller primary particles, and not as a fully dispersed powder. Typical secondary electron contrast highlights the topography and makes it clear that these agglomerates have a rough surface and a relatively open internal structure, with interparticle voids and porosity between subunits, suggesting non-uniform local packing [17,27]. The presence of multiple agglomerates with micrometric dimensions, separated by dark areas, reinforces the idea that the powder has a high tendency to agglomerate, a characteristic frequently associated with the high surface energy of fine ceramic powders and/or processing steps that favor the formation of stable aggregates [28,29]. From a technological standpoint, this scenario is relevant because agglomeration can induce density heterogeneity in the green body and hinder the achievement of a uniform sintered microstructure, especially if the agglomerates are not broken up efficiently during mixing, dispersion, or shaping [30,31,32,33].

When analyzing the region magnified at 15,000×, the microstructure of the agglomerate becomes more evident; thus, it is formed by a stacking of primary particles or sub agglomerates with well-defined contours, exhibiting faceted morphology, which is consistent with the crystalline nature of PZT after calcination [17,34]. From the 5 µm scale bar, it can be seen that the highlighted agglomerate has an overall dimension on the order of a few micrometers, while the constituent units have submicrometric to micrometric dimensions, frequently in the approximate range of ~0.3–1.0 µm. Discontinuities and micro voids are observed between these units, indicating significant intergranular porosity within the agglomerate. Furthermore, the roughness and faceted geometry increase the effective specific area and can intensify particle-particle interactions, reinforce the stability of the agglomerates and increase resistance to dispersion in liquid media [35].

The agglomerated morphology observed in the Sparkler PZT powder is consistent with the behavior typically reported for calcined ferroelectric ceramic powders, where high surface energy promotes particle clustering during drying and storage [17]. Such agglomerates may influence the packing behavior during compaction and the densification kinetics during sintering. According to previous studies on PZT processing, agglomerate fragmentation during pressing is essential to ensure homogeneous packing and to avoid residual porosity in the final microstructure [27,28,29]. If large agglomerates remain intact during compaction, they may generate local density gradients that persist after sintering and act as preferential sites for residual pores, which can negatively affect dielectric and piezoelectric properties due to the interruption of polarization continuity within the ceramic matrix [28,36].

#### 3.1.1. Cluster Size

**Figure 3:**
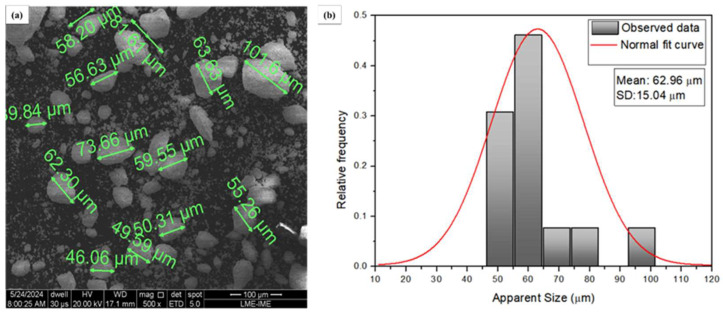
SEM micrograph of PZT-4 Sparkler powder (500×) with apparent size measurements of agglomerates (**a**,**b**) Normal histogram for distribution of sample particle sizes.

Figure 3a presents the measurement of PZT powder agglomerate sizes, highlighting the variability and scale of the particle clustering observed in the material. In turn, Figure 3b shows the corresponding normal distribution histogram of the agglomerate size measurements, providing a statistical representation of the data dispersion, central tendency, and distribution profile of the analyzed particles.

Figure 3a (500×) shows a clearly polydisperse powder, with large particles and aggregates dispersed over a much finer fraction. The measurements annotated in the image for the PZT-Sparkler indicate that the population of larger particles is concentrated mainly in the tens of micrometers range, with values (in µm). With this set, an apparent average size of approximately 62.96 µm is obtained, with a standard deviation of 15.04 µm. The dispersion is relatively high, but this spread is strongly influenced by a much larger value (101.6 µm), which acts as a “tail” for large sizes. In other words, the powder exhibits a dominant population of large particles or aggregates in the 50–60 µm range, accompanied by a smaller fraction of even larger particles. The response that is consistent with the behavior observed in the previous micrographs (Figure 1 and Figure 2), relatively large granules coexisting with fines around them.

Figure 3b shows that the agglomerate size distribution is predominantly concentrated within a narrow intermediate range, as indicated by the higher relative frequencies in the central bins of the histogram. The normal fit curve demonstrates a reasonable agreement with the experimental data, suggesting that the overall distribution can be approximated by a Gaussian profile. However, slight deviations from ideal normality are observed, particularly in the upper size range, where the histogram exhibits a modest right-hand tail. This indicates the presence of a limited number of coarser agglomerates that contribute to the dispersion of the dataset without significantly altering its central tendency. Such behavior reflects a typical partially skewed distribution, where a dominant population governs the material’s microstructural scale, while a secondary fraction of larger agglomerates introduces asymmetry. This distribution pattern is consistent with agglomerated ceramic powders, in which particle clustering and incomplete dispersion lead to a broadened size spectrum and minor departures from perfect statistical symmetry.

#### 3.1.2. Morphological Aspects of Sintered Samples

The evaluation of the morphology of the sintered samples is essential for understanding the relationship between the characteristics of the precursor powder, the microstructural evolution during sintering, and the final functional properties of the ceramic. Parameters such as grain size, grain boundary characteristics, pore distribution, and densification level have a direct influence on ferroelectric domain-wall mobility and, consequently, on the piezoelectric and electromechanical performance of the material.

**Figure 4:**
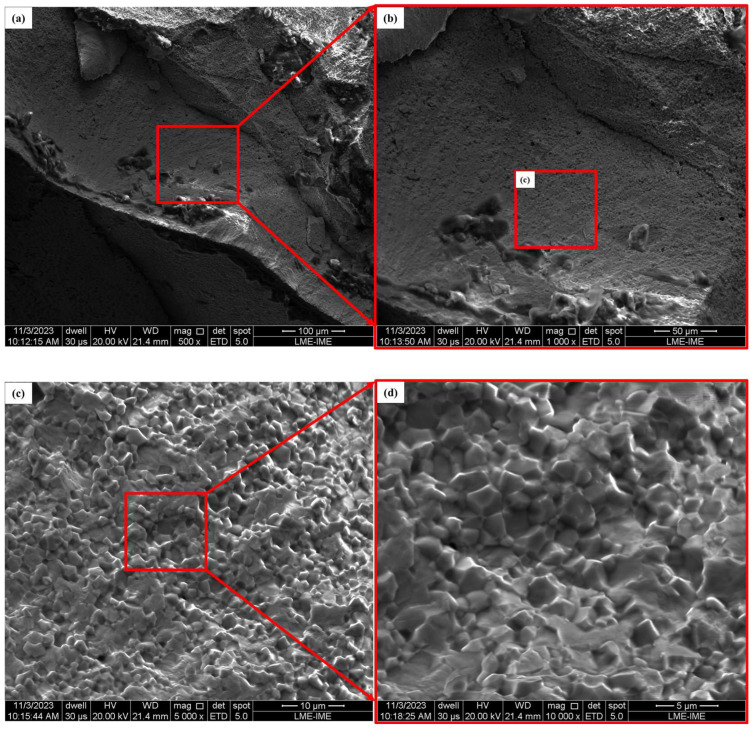
SEM micrographs of the fractured surface of the sintered PZT ceramic at different magnifications: (**a**) 500×; (**b**) 1000×; (**c**) 5000× and (**d**) 10,000×.

The morphology of the fractured surface of the sintered samples was analyzed by scanning electron microscopy (SEM) to investigate the microstructural features developed during the sintering process. Figure 4 presents SEM micrographs acquired at different magnifications from the fractured surface of the sintered PZT ceramic, allowing the evaluation of grain size and morphology, grain boundary characteristics, residual pore distribution, and the degree of microstructural coalescence. Such information is essential for establishing correlations between the morphology of the precursor powder, the microstructural evolution during sintering, and the electromechanical properties observed for the material.

Figure 4 shows SEM micrographs of the fractured surface of the sintered PZT ceramic at different magnifications. At lower magnification, Figure 4a,b reveal a relatively compact fracture surface, with limited evidence of large open pores or extensive microstructural discontinuities. The fracture surface presents regions with different topographic contrast, indicating a heterogeneous fracture path through the sintered ceramic body. The absence of large interconnected pores agrees with the low apparent porosity and high relative density previously obtained by the Archimedes method, suggesting that the sintering treatment promoted effective consolidation of the ceramic.

At higher magnifications, Figure 4c,d reveal a microstructure formed by well-defined micrometric grains with predominantly polygonal and faceted morphology. The grains appear strongly coalesced, with clearly distinguishable grain boundaries and a relatively continuous ceramic network. This morphology indicates that the sintering temperature was sufficient to promote grain growth and interparticle bonding from the agglomerated precursor powder. Although the starting powder exhibited hierarchical agglomerates and interparticle porosity, the sintered body shows a more consolidated microstructure, demonstrating that the thermal treatment effectively reduced the initial powder-related porosity.

The fracture surface also shows the presence of small residual pores, mainly located at intergranular regions and triple junctions between adjacent grains. This pore distribution is typical of conventionally sintered ceramics and indicates that the residual porosity is mostly isolated rather than forming a continuous pore network. Such a microstructural condition is favorable for piezoelectric performance, since isolated and limited porosity reduces dielectric discontinuities and favors a more homogeneous electric-field distribution during polarization. In addition, the presence of well-defined grain boundaries and a dense grain network contributes to more effective ferroelectric domain alignment, which is consistent with the high d_33_ and K_p_ values obtained after polarization.

Therefore, the SEM analysis of the sintered fracture surface establishes a direct correlation between the powder morphology, the sintering process, and the final electromechanical response. The agglomerated morphology of the precursor powder may have contributed to localized residual porosity; however, the high densification achieved after sintering resulted in a compact microstructure with well-developed grains and limited pore concentration. These characteristics help explain the efficient polarization response of the PZT ceramics, particularly under favorable polarization conditionsbetween 100 and 110 °C.

#### 3.1.3. Energy-Dispersive X-Ray Spectroscopy (EDS)

Table 2 presents the chemical composition of the analyzed PZT powder obtained by energy-dispersive X-ray spectroscopy (EDS). The results provide a quantitative assessment of the elemental constituents present in the material, allowing the identification and relative proportion of the main elements associated with the PZT structure.

Table 2 presents the chemical composition of PZT-Sparkler obtained by EDS, in normalized mass %, accompanied by an error value associated with each element. In qualitative terms, the result is consistent with the nature of a Pb(Zr,Ti)O_3_ perovskite-type PZT, since the spectrum clearly identifies the expected elements of the phase: Pb, Zr, Ti, and O. In addition to C, which is normally related to external contributions to the material, as carbon tape, surface contaminants, or organic residues from processing.

The first point that draws attention is the predominance of lead (Pb = 64.72 wt.%), which is expected in mass quantifications by EDS, since Pb has a high atomic number and contributes strongly to the signal, increasing its wt.% fraction when compared to lighter elements. Next come Zr (10.73 wt.%) and Ti (7.10 wt.%), which represent the B site of the perovskite structure. The ratio between Zr and Ti, when analyzed relatively, indicates a material with a higher titanium fraction. Calculating the proportion at site B, we obtain approximately Zr ≈ 0.44 and Ti ≈ 0.56 that is, a composition close to Pb(Zr_0.44_Ti_0.56_)O_3_, which is perfectly plausible for commercial PZTs [36].

The oxygen content (O = 10.85 wt.%) appears relatively low when compared to what would be expected from an ideal Pb(Zr,Ti)O_3_ stoichiometry [36]. Approximately converting the wt.% values to an atomic ratio, the oxygen content is O/(Zr+Ti) ≈ 2.55, while the ideal perovskite would have O/(Zr+Ti) = 3. This difference, however, is very common in EDS and should not be directly interpreted as a real oxygen deficiency for three main reasons. The first EDS has known limitations for light elements, such as O, with greater sensitivity to absorption, geometry. The second point the analysis was performed on powder, with variable topography and effective thickness, which alters the interaction volume and tends to increase uncertainties, and third characteristics the presence of heavy elements (Pb) intensifies absorption in matrix effects that may underestimate O in the quantification. Therefore, the oxygen value should be viewed as semi-quantitative, useful for confirming the presence of the element, but not as a robust measure of stoichiometry.

The presence of carbon (C = 6.59 wt.%) is another relevant point. Therefore, in particulate samples, this C often does not represent an intrinsic part of the PZT, but rather contamination from carbon tape from the sample holder or organic residues from processing. The carbon value, therefore, should be interpreted with caution, especially since the “error” associated with C is significant (10.49%).

The reported errors also deserve attention: Pb (17.52%) and O (14.12%) are relatively high and even carbon shows a high error. This reinforces that the result is excellent for confirming which elements are present and for inferring relative trends, but it has limitations for drawing quantitative conclusions about absolute stoichiometry. In particular, the approximate Pb/(Zr+Ti) ratio ≈ 1.17 suggests an apparent “excess” of Pb. However, this effect may be a direct consequence of underestimation of O, Pb-rich surface segregation in some particles, or typical local variations in powder.

**Figure 5:**
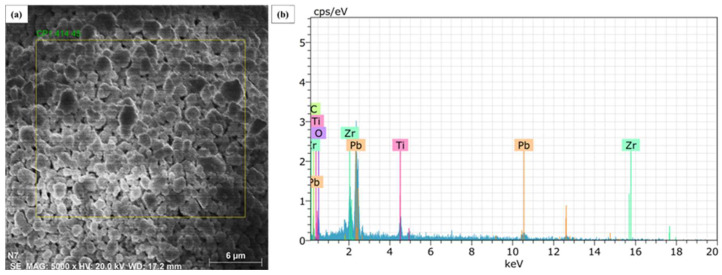
(**a**) SEM micrograph of the PZT-Sparkler (5000×) indicating the area selected for chemical analysis; (**b**) corresponding EDS spectrum, showing the main constituent elements (Pb, Zr, Ti and O), consistent with the typical composition of PZT.

Chemical analysis of the powder was performed by EDS in the region indicated in Figure 5, whose spectrum confirms the presence of the elements Pb, Zr, Ti, and O, consistent with the typical composition of PZT.

The EDS analysis presented in Figure 5 confirms, quite directly, that the sample labeled as PZT-Sparkler contains the characteristic elements of a perovskite-type ceramic Pb(Zr,Ti)O_3_, in addition to a carbon signal which, in most cases, is associated with the preparation analysis environment and not necessarily with the “bulk” composition of the material.

From the spectrum, a set of well-defined peaks attributed to Pb, Zr, Ti, and O can be observed. In the low-energy region (up to ~1 keV), peaks compatible with C (K line, near 0.28 keV), O (K line, ~0.52 keV), and low-energy lines of transition metals appear. This region is particularly useful for confirming the presence of oxygen, but it is also the most sensitive to instrumental uncertainties and absorption effects, which is why EDS is usually semi-quantitative for light elements [37].

In the intermediate range of the spectrum, peaks near ~2 keV are typical of Zr L lines and Pb M lines, which often appear overlapping or very close depending on the detector resolution and correction conditions. Even so, the marking in the spectrum indicating Zr and Pb in this region is consistent with the simultaneous presence of these elements in the PZT. Further on, there is a very characteristic Ti-K peak around ~4.5 keV, which is important because Ti K lines are relatively “clean” and provide robust confirmation of titanium in the sample.

At higher energies, an intense Pb peak is observed around ~10.5 keV, one of the most evident “markers” in PZT due to the high atomic number of lead, which increases the relative intensity of these lines. Finally, the presence of a strong Zr peak in the ~15.7–16 keV range reliably reinforces the identification of zirconium, as high-energy K lines tend to suffer less interference from overlaps with light elements.

Figure 5a shows a granular surface, and the area selected for EDS covers a relatively wide region. However, it is worth remembering that EDS, under these conditions, investigates an interaction volume that can reach the order of micrometers. On a rough and porous surface, this geometry can introduce variations in the electron path and X-ray absorption, mainly affecting the quantification of O. Therefore, even when the elemental identification is solid, the stoichiometry derived from EDS should be treated with caution, especially when discussing “excess or deficiency” of oxygen or lead volatilization based solely on percentages.

**Figure 6:**
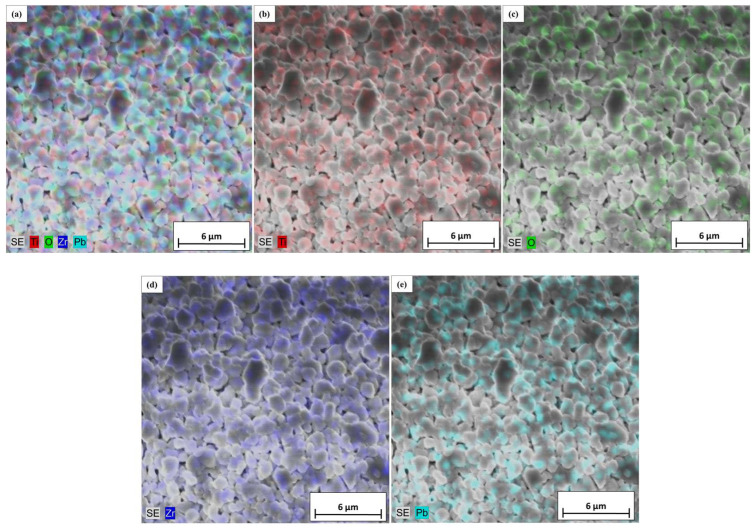
SEM-EDS elemental mapping of PZT-Sparkler powder: (**a**) overlapped elemental distribution of Ti, O, Zr and Pb; (**b**) Ti map; (**c**) O map; (**d**) Zr map; and (**e**) Pb map.

The elemental distribution within the analyzed region is presented in Figure 6, where the overlapped and individual EDS maps reveal a homogeneous spatial distribution of Ti, Zr, Pb, and O, indicating chemical uniformity and absence of detectable elemental segregation.

Chemical mapping by EDS of the PZT-Sparkler sample (Figure 6) allows us to evaluate not only the presence of Ti, Zr, Pb, and O, but also mainly, how these elements are spatially distributed in the analyzed region. In Figure 6a, the mapped area corresponds to a relatively homogeneous granular surface, composed of micrometric particles in contact, with discrete interparticle porosity. This type of morphology is relevant because the EDS signal is influenced by topography: edges, shadows, and pores tend to reduce the detected intensity, especially for light elements such as oxygen.

In general, what is observed in the individual maps is a consistent spatial correlation between Ti (Figure 6b), Zr (Figure 6d), and Pb (Figure 6e), with the concomitant presence of O (Figure 6c), indicating that the analyzed region is dominated by a phase compatible with the perovskite Pb(Zr,Ti)O_3_. In the Ti (Figure 6b) map, the intensity appears disseminated throughout practically the entire solid area, without the formation of isolated intensely enriched “islands” or extensive regions lacking Ti. This suggests that titanium is well incorporated into the ceramic matrix and that there is no evidence, at this scale, of segregation of a Ti-rich phase at a detectable size. Similarly, the Zr (Figure 6d) map also shows a wide and continuous distribution, following the morphology of the particles. The similarity between the Ti and Zr maps is an important indicator of chemical homogeneity at site B of the perovskite structure.

The Pb (Figure 6e) map also shows it distributed throughout the area, confirming that lead is not restricted to specific points but is integrated into the main phase analyzed. In PZT ceramics, it is relatively common to discuss the possibility of surface segregation of Pb-rich compounds or enrichment at grain boundaries, especially after certain thermal cycles. Small local variations in intensity may occur for two main reasons: (i) topographic effects and (ii) the fact that Pb emits intense X-rays, causing subtle changes in geometry and interaction volume to generate apparent contrast. Thus, the most solid interpretation is that Pb is mostly associated with the same microstructure that contains Zr and Ti, reinforcing the dominance of the PZT phase in the analyzed region.

The oxygen map (Figure 6c) shows, as expected, a signal more sensitive to morphology, where regions of porosity and interparticle gaps tend to appear with lower intensity, and even in solid areas there may be variations related to absorption effects and the local geometry of X-ray emission and detection. It is important to emphasize that, although the O map confirms the presence of oxygen and its association with the particles, the absolute quantification of oxygen by EDS is intrinsically less robust than that of Pb, Zr, and Ti, especially on rough surfaces and at an accelerating voltage of 20 kV, because the oxygen signal involves low-energy X-rays and is more strongly affected by matrix corrections. From a processing–structure–property perspective, the agglomerated morphology observed in the powder may influence particle packing during compaction and contribute to local densification heterogeneities after sintering. Such heterogeneities may generate residual porosity and localized variations in electric-field distribution during polarization, potentially affecting ferroelectric domain alignment and, consequently, the electromechanical response of the material. However, the high relative density obtained after sintering (96.02%) together with the elevated d_33_ and K_p_ values indicate that the influence of these microstructural features was effectively minimized under the processing conditions adopted in this study.

### 3.2. Physical Characterization

Table 3 presents the physical characterization results of the sintered PZT powder samples obtained by the Archimedes method, including submerged mass, wet mass, dry mass, water absorption, apparent porosity, apparent density, and relative density. These parameters provide a comprehensive evaluation of the material’s densification behavior and porosity level after sintering, enabling a detailed assessment of the consolidation efficiency and structural integrity of the samples.

Analysis of the results presented in Table 3 indicates that the PZT sintered sample exhibits physical behavior consistent with a well-processed and adequately sintered ceramic material. The submerged, wet, and dry masses show very similar values with low standard deviations, demonstrating good experimental repeatability and suggesting a structure with a reduced volume of open pores. The small difference observed between the wet and dry masses indicates that the sample absorbs a minimal amount of water, corroborating the low connectivity of the porous network.

This behavior is confirmed by the water absorption value, which remained at 0.12 ± 0.08%, considered quite low for PZT-based ceramics. This result indicates that the microstructure has few interconnected pores, a typical characteristic of materials subjected to an efficient sintering process. Low water absorption is a particularly relevant aspect for piezoelectric applications, since the presence of moisture can negatively affect the dielectric properties and functional stability of the material.

The apparent porosity, determined to be 0.91 ± 0.66%, reinforces this interpretation, showing that only a small fraction of the total material volume corresponds to pores accessible to the fluid. Although the standard deviation associated with this parameter is relatively higher, suggesting some local microstructural heterogeneity, the average value obtained still indicates a globally well-densified material. This heterogeneity may be associated with variations in powder compaction or thermal gradients during sintering.

The apparent density of 7.68 ± 0.06 g/cm^3^ is high and consistent with the expected values for PZT ceramics, indicating that most of the material’s volume is effectively occupied by the solid phase [27]. The low dispersion of this result points to good control of the manufacturing process, reflecting uniformity in the forming and heat treatment steps. Consequently, the relative density of 96.02 ± 0.87% demonstrates that the sample reached a high degree of densification in relation to the theoretical density of the material, leaving only a limited fraction of pores, predominantly closed or isolated.

The relative density obtained (~96%) is within the typical range reported for conventionally sintered PZT ceramics, which generally varies between 92% and 98% depending on the powder characteristics and sintering parameters [27,28]. Achieving densities above 90% is considered essential for obtaining stable piezoelectric properties, since residual porosity can significantly reduce the effective dielectric permittivity and limit the mobility of ferroelectric domain walls. Therefore, the densification level obtained in the present study is sufficient to ensure reliable electromechanical performance, although further improvements in powder dispersion or sintering optimization could potentially reduce the residual porosity fraction and further enhance the functional response.

### 3.3. X-Ray Diffraction Analysis

**Figure 7:**
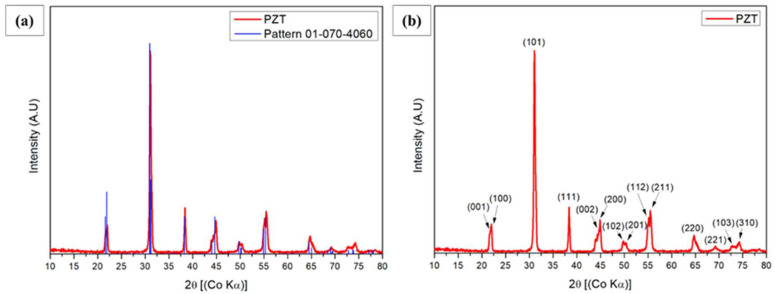
(**a**) Comparison of the diffractogram of PZT card 01-070-4060 and Sparkler Ceramics PZT powder and (**b**) Indexing of the crystalline planes (hkl) referring to the evaluated PZT sample.

Figure 7a shows the diffraction pattern obtained for the PZT sample evaluated in this study in relation to crystallographic card number 01-070-4060, with the respective values of relative intensity and diffraction angle (2θ) observed for the PZT sample pattern (in blue) compared to the diffractogram obtained for the evaluated samples (red). Figure 7b shows the diffraction pattern of the PZT sample with the respective indexed planes.

Figure 7a,b provide important information about the crystalline structure of the analyzed PZT sample, allowing for the evaluation of both the correspondence with the reference standard and the crystallographic organization of the planes responsible for the main reflections. In general, the comparison presented in Figure 7a indicates good agreement between the experimental diffractogram of the sample and catalog card 01-070-4060, since the angular positions of the main peaks and the overall distribution of relative intensities show high compatibility with the expected pattern for the PZT phase. This agreement is a strong indication that the obtained material preserves the characteristic crystalline structure of the perovskite phase, without significant peak shifts or marked appearance of additional reflections that would clearly suggest the predominance of secondary phases. Furthermore, the good definition of the peaks, associated with the low background level of the diffractogram, suggests that the sample has a high degree of crystallinity, with limited amorphous contribution.

Another relevant aspect to highlight is that, although there is a good correspondence between the experimental standard and the reference data sheet, small differences in the relative intensities between some peaks can be observed. This behavior is common in polycrystalline ceramic materials and may be associated with factors such as preferred grain orientation, microstructural variations resulting from processing, differences in compaction, crystallite size, residual microstrains, and even small local compositional variations. In PZT systems, these changes in relative intensity do not necessarily indicate a phase change, but may reflect modifications in the statistical distribution of crystallographic orientations or in the degree of microstructural development after sintering.

Figure 7b, in turn, complements this analysis by presenting the experimental diffractogram with indexed crystallographic planes, allowing direct identification of the reflections associated with the material’s structure. The indexing of these planes confirms that the material possesses an organized and coherent crystalline lattice consistent with the perovskite structure expected for PZT. The most intense peak is associated with the (101) plane, located in the region near 30° in 2θ, indicating that this reflection constitutes the main diffracted contribution of the sample. The high intensity and small apparent width of this peak suggest good crystalline ordering and adequate growth of the crystalline regions.

From a microstructural viewpoint, the sharpness of the peaks observed in Figure 7a,b suggests that the sample exhibits a good level of crystallinity and that the heat treatment employed was sufficient to promote the development of the desired crystalline phase. The absence of excessive peak broadening indicates, qualitatively, that the material is neither highly nanocrystalline nor strongly strained, although a quantitative conclusion about the crystallite size and microstrain requires specific analyses, such as the Scherrer method or a complete structural refinement. Even so, qualitatively, the observed pattern is consistent with a well-crystallized and structurally stable ceramic material.

Table 4 summarizes additional crystallographic information extracted from the reference database card used for phase identification of the PZT samples. The table includes structural parameters such as empirical and chemical formulas, crystal system, space group, unit cell dimensions, theoretical density, and the main diffraction peaks with their respective interplanar spacing’s, diffraction angles, and relative intensities. These data are essential for supporting phase identification and for correlating the experimental XRD results with the expected crystallographic structure of the material.

The crystallographic data indicate that the analyzed PZT corresponds to a lead zirconate titanate composition with empirical formula O_3_PbTi0.48Zr0.52 and chemical formula Pb(Zr0.52Ti0.48)O_3_, consistent with compositions commonly associated with PZT materials in the vicinity of the morphotropic phase boundary region reported in the literature. The material was indexed in the tetragonal crystal system, belonging to the space group P4mm, characteristic of ferroelectric PZT compositions with spontaneous polarization along the c-axis. The unit cell parameters (a = b = 4.0550 Å and c = 4.1100 Å) result in a tetragonality ratio c/a ≈ 1.014, indicating a slight tetragonal distortion associated with the non-centrosymmetric structure required for piezoelectric activity.

**Figure 8:**
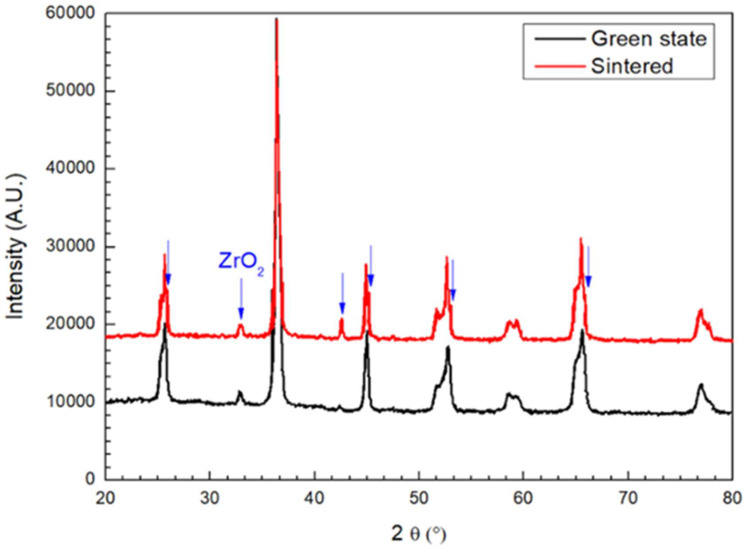
Diffraction patterns of PZT powder and sintered samples.

Figure 8 compares the X-ray diffraction patterns of the PZT samples in the green state prior to sintering (black curve) and after sintering (red curve). This comparison allows the evaluation of the structural evolution promoted by the thermal treatment, highlighting changes in crystallinity, peak definition, phase development, and the possible persistence or reduction in secondary phases throughout the densification process.

Analysis of the X-ray diffractograms obtained for PZT-Sparkler in the green state and after sintering clearly shows the effect of heat treatment on the crystalline structure and the degree of ordering of the material. In both cases, the patterns are dominated by reflections characteristic of the perovskite phase Pb(Zr,Ti)O_3_, indicating that the main phase is already formed in the green state and is preserved after sintering. However, marked differences are observed in the intensity, width, and definition of the peaks, reflecting microstructural changes induced by the thermal process.

In the green state, the diffraction peaks exhibit lower intensity and greater broadening, as well as a relatively higher background, which is consistent with a material composed of smaller crystallites, greater microstrain, and a higher degree of structural disorder. This behavior is typical of compacted bodies before densification, in which crystalline coherence is limited and any local heterogeneities have not yet been eliminated. After sintering, a significant increase in peak intensity is observed, accompanied by a clear reduction in width at half-height, indicating growth of coherent diffraction domains, relaxation of internal stresses, and an overall increase in crystallinity. This result is in full agreement with the densification observed experimentally and with the formation of a more stable and ordered microstructure.

The most intense peaks of the sintered diffractogram, distributed along the 2θ interval between 20° and 80°, correspond to the typical reflections of the PZT perovskite structure, considering the Co-Kα radiation used. In specific regions of the diffractogram, particularly in the range associated with pseudocubic reflections around 45° and 52–53°, an asymmetry or beginning of peak splitting is observed, more clearly resolved in the sintered sample. This behavior suggests that the crystal structure is not perfectly cubic and is compatible with a ferroelectric distortion associated with the tetragonal symmetry identified by the Rietveld refinement. Although compositions near the morphotropic phase boundary may exhibit coexistence of tetragonal and rhombohedral features, no direct phase coexistence or domain structure analysis was performed in the present study.

In addition to the perovskite phase, the diffractogram reveals the presence of low-intensity reflections attributed to the secondary phase ZrO_2_, highlighted in the region around 33° and in other specific positions indicated on the graph. The detection of this phase indicates that, although perovskite is dominant, a residual fraction of ZrO_2_ persists even after sintering. The presence of this phase may be associated with an incomplete reaction during synthesis or, alternatively, with segregation processes induced by partial volatilization of PbO at high temperatures, leading to locally enriched zirconium regions. The fact that the reflections attributed to ZrO_2_ are relatively weak suggests that this phase is present in a low volume fraction, which is consistent with the good densification and overall electromechanical performance observed. Nevertheless, their quantification is relevant, since non-ferroelectric secondary phases can act as functional discontinuities, influence dielectric, and mechanical losses, especially if they are heterogeneously distributed.

From an electromechanical standpoint, trace amounts of ZrO_2_ may locally affect the polarization process by acting as non-ferroelectric inclusions within the ceramic matrix, potentially reducing domain-wall mobility and introducing localized dielectric discontinuities. Such effects may contribute to additional dielectric or mechanical losses when secondary phases are present in significant fractions or distributed heterogeneously. However, in the present study, the low intensity of the ZrO_2_ reflections together with the high d_33_ and K_p_ values obtained indicate that the residual secondary phase fraction was insufficient to significantly impair the overall piezoelectric and electromechanical performance of the PZT ceramics.

**Figure 9:**
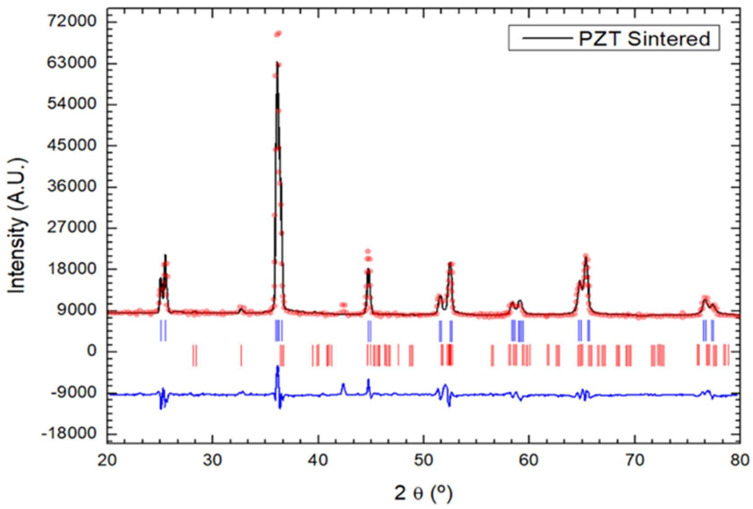
Rietveld refinement of the X-ray diffraction pattern of the sintered PZT sample. Experimental diffraction data are represented by red circles, the calculated pattern by the black solid line, and the difference curve (Yobs − Ycalc) by the blue line. Vertical markers indicate the Bragg reflection positions of the crystalline phases considered in the refinement, including the perovskite Pb(Zr,Ti)O_3_ phase and minor ZrO_2_ reflections.

The better definition of these characteristics in the sintered material reflects the higher crystalline quality and reinforces the need for quantitative analysis by Rietveld refinement for the precise identification of the crystallographic phases present and their relative fractions, as shown in Figure 9.

Figure 9 shows the X-ray diffractogram of the sintered PZT sample accompanied by the Rietveld method fitting, allowing for a more in-depth and quantitative analysis of the material’s crystalline structure [38,39]. The excellent visual alignment between the experimental points (red symbols) and the calculated pattern (solid black line) indicates that the adopted structural model satisfactorily describes the experimental data throughout the entire investigated angular range (20° ≤ 2θ ≤ 80°), demonstrating the high quality of the refinement.

The observed diffraction peaks exhibit high intensity and reduced width at half-height, confirming the high degree of crystallinity of PZT after sintering, in agreement with the high densification previously determined by the Archimedes method. The most intense peak, located in the region around 36–37°, along with the other reflections distributed throughout the diffractogram, is characteristic of the perovskite phase Pb(Zr,Ti)O_3_, reinforcing that this is the dominant crystalline phase in the sintered material. The good definition of the peaks in this region is particularly relevant, as it reflects the growth of crystalline domains and the reduction in internal microstrains induced by the sintering thermal cycle [39].

The distribution of vertical markers associated with the phases considered in the refinement shows excellent correspondence with the positions of the experimental maxima, indicating that the observed reflections are adequately explained by the set of phases included in the model. The high density of markers in the central region of the diffractogram is typical of the perovskite structure of PZT and confirms the correct indexing of the principal reflections [39,40,41]. The possible presence of additional low-intensity markers suggests the inclusion of secondary phases in a reduced fraction, consistent with previous qualitative observations of ZrO_2_ traces, whose contribution is small and does not compromise the overall fit.

The difference curve (represented in the lower part of the graph, in blue) remains close to the baseline throughout the entire 2θ interval, showing only residual oscillations of low amplitude, mainly in the regions of more intense peaks. This behavior is typical of successful refinements, in which small discrepancies can be attributed to instrumental effects, residual peak asymmetries, discrete preferential microtexture, or small local variations in composition [13,40]. The absence of relevant systematic deviations in the difference curve indicates that there are no important unmolded crystalline phases, nor significant structural flaws in the adopted refinement.

From a crystallographic point of view, the quality of the fit obtained reinforces that the sintered PZT exhibits a well-developed perovskite structure, with lattice parameters and crystal symmetry adequately described by the refined model [41]. The good resolution of the reflections, especially in symmetry-sensitive regions, provides a solid basis for the precise identification of the predominant ferroelectric phase and for the eventual quantification of phase coexistence, if applicable. This aspect is particularly relevant for PZTs near the morphotropic boundary, in which small structural variations have a direct impact on functional properties.

The predominance of the perovskite Pb(Zr,Ti)O_3_ phase confirms the successful stabilization of the ferroelectric structure during sintering. The presence of minor ZrO_2_ reflections has been reported in several studies involving commercial PZT powders and is frequently associated with slight deviations from ideal stoichiometry or partial volatilization of PbO during high-temperature processing [38,39,40,41,42]. Although the detected fraction appears to be small, secondary phases can locally influence dielectric losses and mechanical damping. Nevertheless, the strong dominance of the perovskite phase indicates that the crystallographic structure responsible for the piezoelectric response is well preserved.

### 3.4. Electromechanical Properties Characterization

Table 5 presents the electromechanical properties obtained for the PZT samples. The reported data provide a comprehensive evaluation of the material’s functional performance, including key parameters related to piezoelectric response, dielectric behavior, and electromechanical coupling. These results are essential for assessing the efficiency of the polarization process and the overall performance of the material, as well as for establishing correlations between processing conditions, microstructure, and functional properties.

Table 5 shows that the electromechanical properties of the sintered PZT samples were significantly influenced by the polarization conditions, particularly time and temperature, since the electric field was kept constant at 3.0 kV/mm for all conditions.

**Figure 10:**
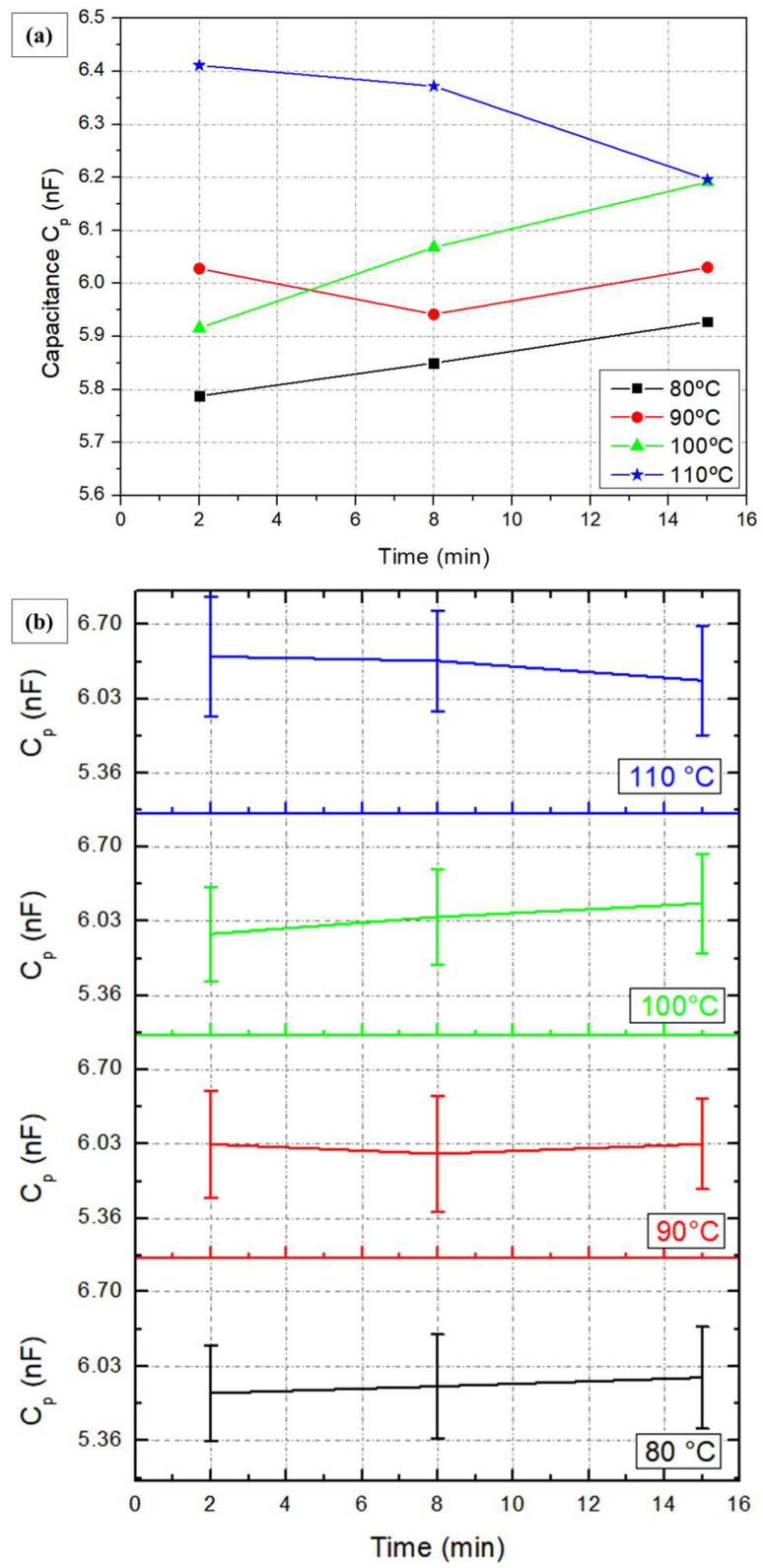
Capacitance (C_p_) behavior of PZT samples as a function of polarization time under different temperatures. (**a**) Clustered curves and (**b**) Average values with standard deviations.

Figure 10 presents the capacitance (C_p_) results obtained for the PZT samples as a function of polarization time at different temperatures (80, 90, 100, and 110 °C). The graphs allow a direct visualization of the influence of the processing parameters on the dielectric response of the material, including the dispersion of the measured values represented by the error bars.

The trends observed in Figure 10 are consistent with the numerical values previously summarized in Table 5. In general, the capacitance (C_p_) remained within a relatively narrow range for all polarization conditions, although measurable variations were observed as a function of polarization time and temperature, indicating that this parameter is also sensitive to the processing conditions. The C_p_ values ranged from 5.788 ± 0.430 nF to 6.411 ± 0.538 nF; whereas the piezoelectric coefficient d_33_ and the electromechanical coupling factor K_p_ exhibited more pronounced changes among the samples. The d_33_ values varied from 241.7 ± 11.1 to 325.8 ± 3.9 pC/N, while K_p_ ranged from 0.416 ± 0.016 to 0.509 ± 0.006. These results indicate that the polarization parameters affected all evaluated properties; however, their influence was more significant on ferroelectric domain alignment and electromechanical conversion than on the dielectric response of the material.

Regarding capacitance, the values remained within a relatively narrow range, although clear variations were observed among the different polarization conditions, indicating that the dielectric response of the material was also affected by the processing parameters. The highest capacitance was observed for sample SS-2/110/3 (6.411 nF), followed by SS-8/110/3 (6.372 nF) and SS-15/100/3 (6.191 nF), while the lowest value was recorded for SS-2/80/3 (5.788 nF). These results suggest that both temperature and polarization time influenced the dielectric behavior, likely through changes in domain wall mobility, polarization state, and internal dipole alignment. Although the variation in C_p_ was smaller than that observed for d_33_ and K_p_, the capacitance still proved to be responsive to the applied polarization conditions and therefore represents an additional useful parameter for evaluating the functional evolution of the PZT samples.

**Figure 11:**
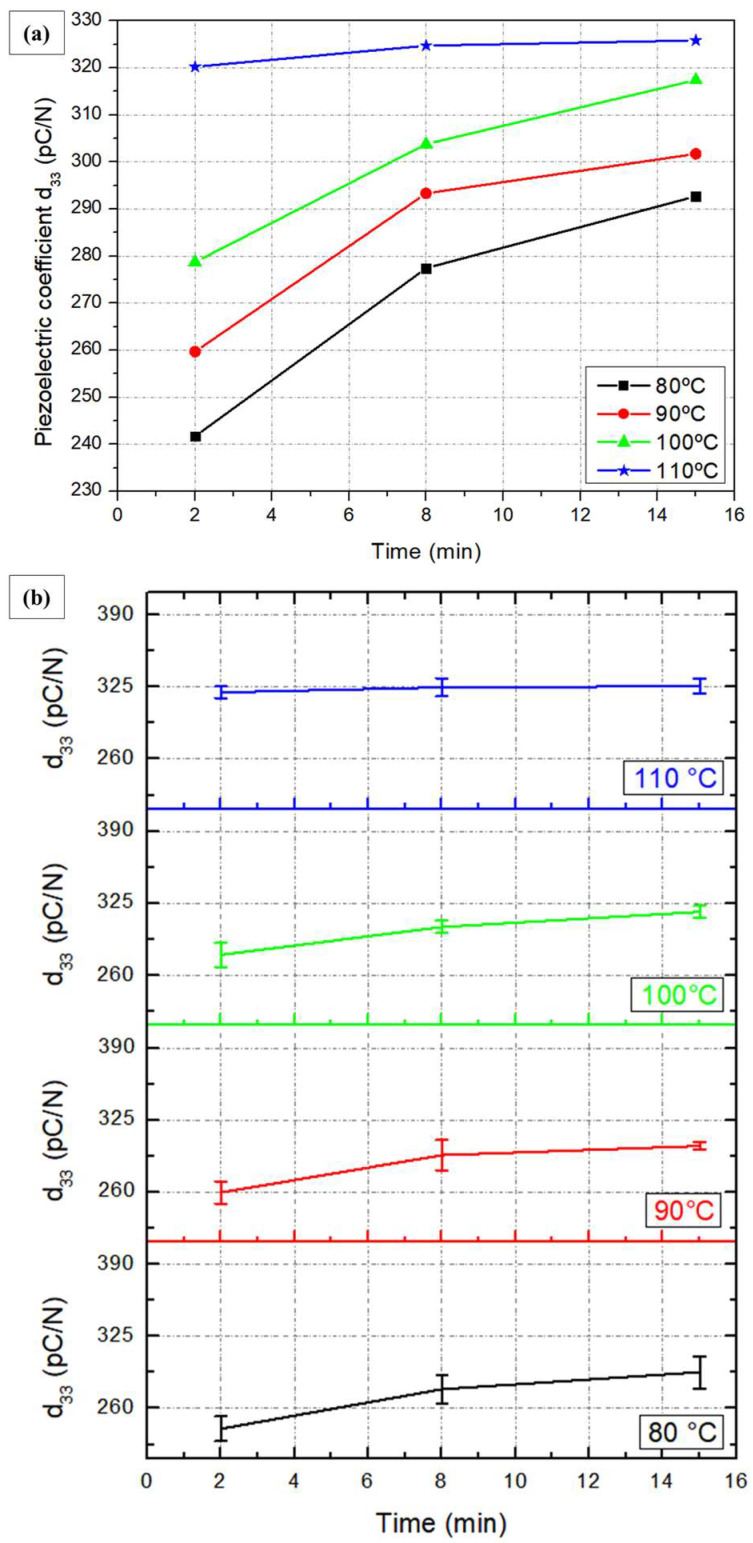
Piezoelectric coefficient (d_33_) behavior of PZT samples as a function of polarization time under different temperatures. (**a**) Clustered curves and (**b**) Average values with standard deviations.

Figure 11 presents the variation in the piezoelectric coefficient (d_33_) of the PZT samples as a function of polarization time for the different temperatures evaluated (80, 90, 100, and 110 °C). The graphs provide a direct comparison of the influence of thermal and temporal polarization conditions on the domain alignment efficiency, with the error bars indicating the dispersion of the experimental measurements.

The behavior shown in Figure 11 is consistent with the values reported in Table 5, confirming that d_33_ was strongly affected by the polarization parameters. The piezoelectric coefficient d_33_ exhibited the largest variation among the analyzed properties, ranging from 241.7 ± 11.1 to 325.8 ± 3.9 pC/N. This result highlights the strong influence of polarization conditions on the degree of ferroelectric domain alignment along the 33 direction. The highest d_33_ value was obtained for sample SS-15/110/3 (325.8 ± 3.9 pC/N), followed closely by SS-8/110/3 (324.7 ± 4.2 pC/N) and SS-15/100/3 (317.4 ± 5.3 pC/N), indicating that elevated temperatures combined with intermediate or longer polarization times were the most effective conditions for promoting domain orientation and maximizing the piezoelectric response. Conversely, the lowest value was observed for SS-2/80/3 (241.7 ± 11.1 pC/N), suggesting that the combination of lower temperature and shorter polarization time was insufficient to achieve effective domain alignment.

A comparison at constant temperature further supports this interpretation. At 80 °C, a progressive increase in d_33_ with polarization time is observed, rising from 241.7 ± 11.1 pC/N at 2 min to 277.4 ± 12.8 pC/N at 8 min and reaching 292.7 ± 14.6 pC/N at 15 min. This trend indicates that, under lower thermal activation, longer polarization times are required to promote continued domain reorientation and stabilization of the polarized state. In other words, the polarization kinetics at 80 °C appear to be slower, making exposure time a decisive factor for improving the piezoelectric response. The gradual increase also suggests that saturation was not fully achieved within the shortest treatment times under this temperature condition.

However, this monotonic behavior is not maintained at higher temperatures. At 90 °C, the sample polarized for 2 min exhibited a d_33_ value of 259.7 ± 10.2 pC/N, while longer polarization times increased the piezoelectric response to 293.3 ± 13.6 pC/N and 301.7 ± 3.4 pC/N for 8 and 15 min, respectively. This behavior suggests that, at this temperature, the initial polarization stage is already highly effective due to the increased mobility of domain walls, allowing rapid dipole alignment within a short treatment time. The reduction observed at 8 min, followed by partial recovery at 15 min, indicates that the effect of time is non-linear and may involve competing phenomena such as temporary stabilization of unfavorable domain configurations, local relaxation processes, or progressive saturation of the polarization process. Therefore, 90 °C appears to represent an intermediate condition in which efficient alignment can be achieved rapidly, but prolonged exposure does not necessarily lead to proportional gains in piezoelectric performance.

At 100 °C, the d_33_ values were 278.7 ± 10.9, 303.8 ± 5.5, and 317.4 ± 5.3 pC/N for 2, 8, and 15 min, respectively. Under this condition, increasing the polarization time promoted a progressive enhancement of the piezoelectric response, indicating that longer polarization times favored domain alignment and stabilization. The increase from 2 to 8 min was particularly significant, whereas the additional increase observed at 15 min suggests that the material continued to approach a more saturated polarization state at this temperature. These results indicate that 100 °C provided an effective thermal condition for promoting efficient domain switching and progressive polarization enhancement, although the highest overall d_33_ values were obtained at 110 °C. A similar tendency was observed for K_p_, reinforcing the existence of an optimal polarization window rather than a simple linear dependence between processing time and functional enhancement.

For 110 °C, the d_33_ values were 320.2 ± 5.2 pC/N at 2 min, 324.7 ± 4.2 pC/N at 8 min, and 325.8 ± 3.9 pC/N at 15 min. In contrast to the behavior observed at 90 and 100 °C, a gradual increase with polarization time is verified, although less pronounced than that observed at 80 °C. The small difference between 8 and 15 min suggests that the system approaches saturation under these conditions, with only marginal gains at longer times. Therefore, The highest d_33_ values were obtained at 110 °C, indicating efficient domain alignment under elevated thermal activation.

**Figure 12:**
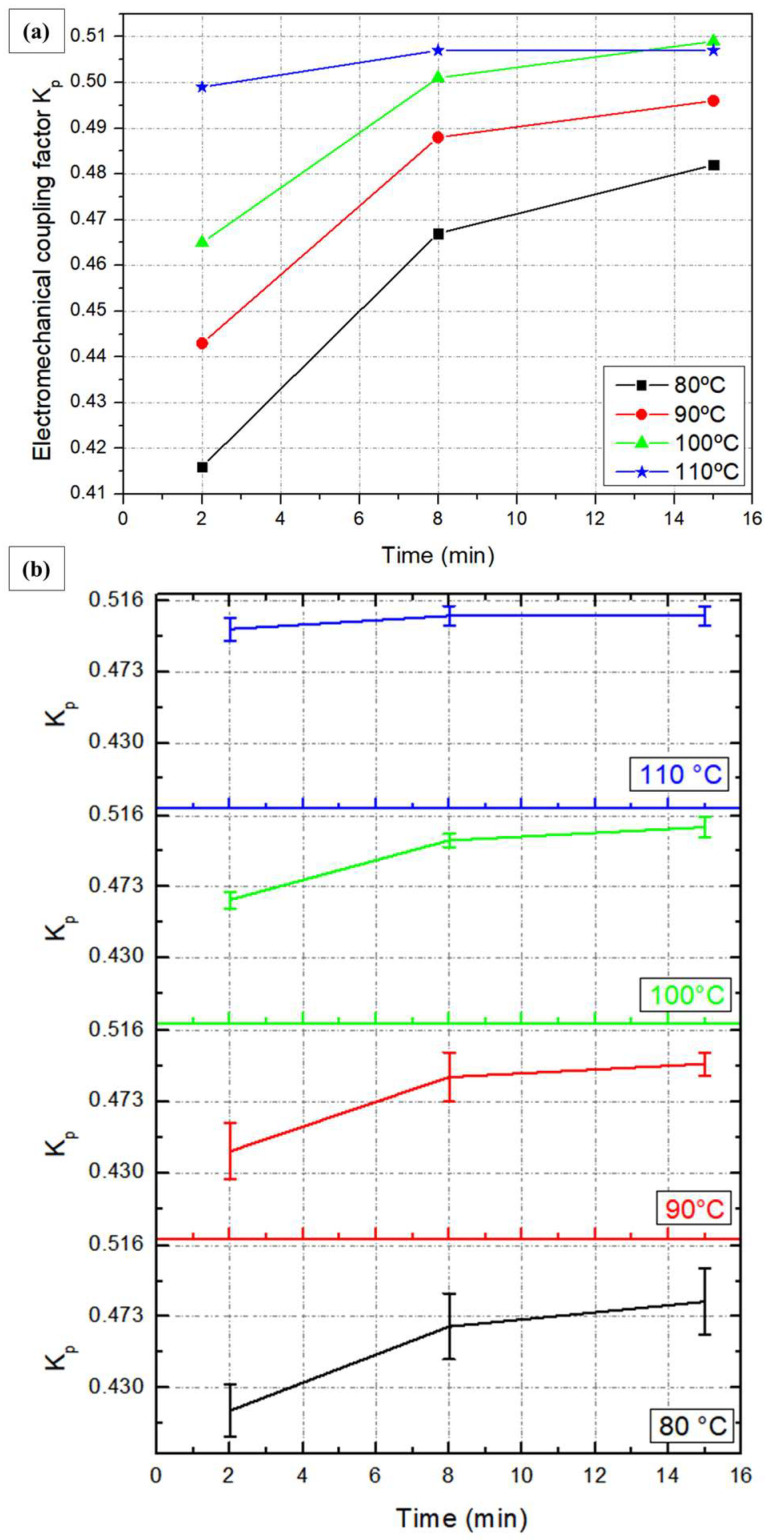
Electromechanical coupling factor (K_p_) behavior of PZT samples as a function of polarization time under different temperatures. (**a**) Clustered curves and (**b**) Average values with standard deviations.

Figure 12 presents the variation in the electromechanical coupling factor (K_p_) of the PZT samples as a function of polarization time for the different temperatures investigated (80, 90, 100, and 110 °C). The graphs enable the visualization of how the polarization conditions affect the efficiency of energy conversion between electrical and mechanical domains, while the error bars represent the experimental variability of the measurements.

The tendencies observed in Figure 12 are in agreement with the values summarized in Table 5, confirming that K_p_ was sensitive to both polarization time and temperature. The electromechanical coupling factor K_p_ followed a general trend similar to that of d_33_, as expected, since both properties are directly related to the efficiency of electromechanical energy conversion. The values ranged from 0.416 ± 0.016 to 0.509 ± 0.006. The lowest value was observed for sample SS-2/80/3, while the highest values were obtained for SS-15/100/3 (0.509 ± 0.006), SS-8/110/3 (0.507 ± 0.006), and SS-15/110/3 (0.507 ± 0.006). These results indicate that the highest electromechanical coupling values were predominantly achieved at elevated polarization temperatures, particularly between 100 and 110 °C, where domain mobility and polarization efficiency were more favorable. Longer polarization times also contributed to improving the coupling response, although their influence became less pronounced at higher temperatures due to the tendency toward polarization saturation. This behavior suggests that electromechanical coupling benefits not only from enhanced domain alignment, but also from improved interaction between the electrical and mechanical responses of the ceramic under optimized polarization conditions.

A combined analysis of d_33_ and K_p_ indicates that the optimal polarization condition depends on the targeted performance criterion. If the objective is to maximize the longitudinal piezoelectric coefficient, the most favorable condition corresponds to 110 °C for 15 min, which produced the highest d_33_ value (325.8 ± 3.9 pC/N), closely followed by 110 °C for 8 min and 100 °C for 8 min. On the other hand, if the focus is on maximizing the electromechanical coupling factor, the best performance was obtained under conditions between 100 and 110 °C, particularly for 8 and 15 min, where the highest K_p_ values were recorded. This distinction demonstrates that different functional properties may respond differently to the same processing parameters, reflecting the complex relationship between domain alignment, dielectric behavior, and electromechanical conversion in ferroelectric ceramics. Therefore, the selection of polarization conditions should consider the specific property to be optimized for the intended application.

From a scientific perspective, this behavior is consistent with the existence of an optimal polarization window, in which the balance between domain wall mobility, dipole alignment, process saturation, and possible relaxation mechanisms leads to maximum functional performance. Therefore, the data in Table 5 indicate that the electromechanical properties of sintered PZT can be significantly optimized through careful adjustment of polarization conditions, with particularly promising results observed between 100 and 110 °C and polarization times between 8 and 15 min, depending on the targeted property. These findings also provide a solid experimental basis for the application of kinetic models, such as the Avrami approach, enabling a quantitative correlation between the evolution of domain alignment and the processing parameters employed.

It should be emphasized that the present conclusions are restricted to the investigated polarization window, in which the electric field was maintained constant at 3.0 kV/mm. Therefore, the observed trends reflect the combined influence of polarization temperature and time under a fixed-field condition and should not be interpreted as a complete optimization of all polarization parameters. Additional studies involving simultaneous variation in electric field, temperature, and polarization time would be required to establish a broader polarization-processing map for the investigated PZT-4 ceramics.

Table 6 presents the Avrami parameters obtained from the kinetic fitting of the domain alignment process in the PZT samples, considering the variation in polarization time and temperature. The table includes the experimental d_33_ values, the normalized transformed fraction Y(t), and the Avrami parameters k and n, allowing the evaluation of how the polarization conditions affect the evolution of domain orientation along the 33 direction.

The results indicate that the transformed fraction Y(t), estimated from the ratio between the experimental d_33_ and the reference value adopted for full alignment, increases progressively with polarization time for all temperatures evaluated. This behavior confirms that longer polarization times promote a higher fraction of domains oriented along the 33 direction. At 80 °C, Y(t) increases from 0.73 to 0.89, indicating a slower alignment process and suggesting that this temperature provides limited thermal activation for domain reorientation.

At 90 °C, the transformed fraction increases from 0.79 to 0.91, showing improved polarization efficiency compared with 80 °C. This suggests that the increase in temperature facilitates domain wall mobility and accelerates the alignment process. A similar tendency is observed at 100 °C, where Y(t) rises from 0.84 to 0.96, indicating a more advanced stage of domain alignment after 15 min.

The highest fractions are observed at 110 °C, with Y(t) values ranging from 0.97 to 0.99. This indicates that, under this temperature condition, the polarization process approaches saturation even at short times. The Avrami analysis indicated faster apparent alignment kinetics at higher temperatures.

The apparent fitting parameter k obtained from the Avrami equation increased with temperature, indicating that the experimental d_33_ evolution curves approached their saturation levels more rapidly at higher polarization temperatures. Within the phenomenological framework adopted in this work, this parameter should be interpreted only as a descriptor of the fitting behavior and not as a true physical kinetic constant associated with nucleation-and-growth processes.

The apparent Avrami exponent n remained relatively low throughout the investigated temperature range and exhibited modest variations among the fitting conditions. Because the Avrami model is employed here solely as a phenomenological fitting approach, no mechanistic interpretation is assigned to these variations. The exponent should therefore be viewed only as a mathematical descriptor related to the overall shape of the polarization-evolution curves.

Overall, the data show that temperature has a stronger influence than time on the polarization kinetics of the PZT samples. While increasing time promotes gradual domain alignment, increasing temperature accelerates the process and leads to higher transformed fractions. The combination of high k, low n, and Y(t) values close to unity at 110 °C indicates that this condition produces the fastest and most complete domain alignment among the evaluated groups.

**Figure 13:**
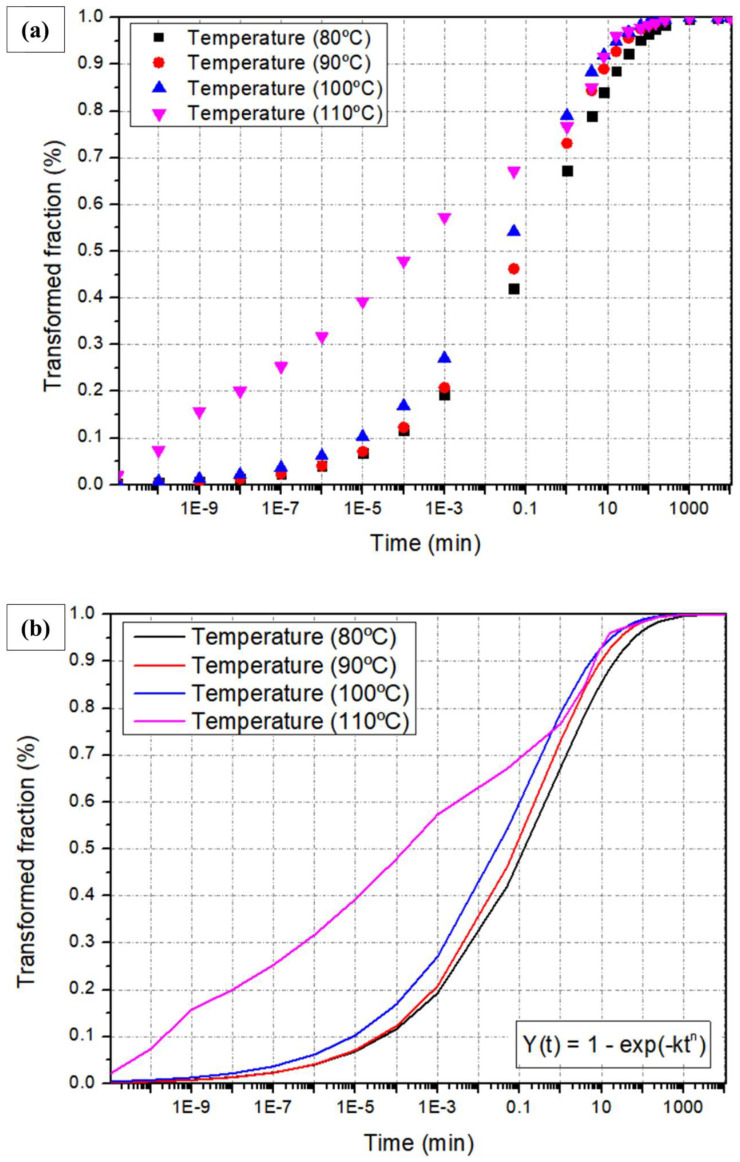
Avrami kinetic analysis of the polarization process in PZT samples under different temperature conditions. (**a**) Experimental transformed fraction, Y(t), calculated from the normalized d_33_ values as a function of polarization time. (**b**) Avrami fitting curves obtained using the apparent kinetic parameters k and n, showing the evolution of the apparent domain alignment process at different polarization temperatures.

Figure 13 presents the Avrami kinetic curves obtained for the PZT samples under different polarization temperatures. Figure 13a shows the transformed fraction values calculated from the experimental data, while Figure 13b presents the corresponding fitted curves generated from the Avrami model using the kinetic parameters k and n. These graphical representations allow a direct comparison of the domain alignment kinetics as a function of polarization time and temperature.

The kinetic curves presented in Figure 13 indicate a progressive evolution toward saturation behavior with increasing polarization time and temperature. For the 80–100 °C conditions, lower transformed fraction values were observed at shorter polarization times, suggesting that domain alignment initially occurred more gradually under limited thermal activation. As the polarization time increased, the transformed fraction progressively approached higher values, indicating enhanced domain reorientation and stabilization. In particular, the 110 °C condition exhibited transformed fraction values close to saturation even at short polarization times, confirming the higher apparent kinetic response previously observed for this condition.

Although the Avrami formalism was employed only as a phenomenological approximation, the fitted curves adequately represented the overall tendency of the experimental data. The results suggest that increasing temperature not only accelerated the apparent polarization kinetics but also reduced the time required to approach a highly aligned ferroelectric state. Furthermore, the convergence of the curves at longer polarization times indicates that all investigated conditions tended toward similar final alignment levels, with the main difference being the kinetic path required to reach this state.

### 3.5. Statistical Analysis

Table 7 presents the two-way ANOVA results for capacitance (C_p_), considering polarization temperature, polarization time, and the interaction between these two factors as independent variables.

The ANOVA results indicate that capacitance was not significantly affected by the polarization conditions within the investigated experimental range. The effect of polarization temperature showed a *p*-value of 0.05827, which is slightly above the adopted significance level of *p* ≤ 0.05. Although this result suggests a possible tendency for temperature to influence C_p_, the effect cannot be considered statistically significant. In contrast, polarization time exhibited a much higher *p*-value (*p* = 0.94073), indicating that increasing the polarization time from 2 to 15 min did not produce a relevant change in capacitance. Similarly, the interaction between temperature and time was not significant (*p* = 0.94160), demonstrating that the combined variation in these parameters did not substantially modify the dielectric response of the material.

These findings suggest that C_p_ remained comparatively stable under the evaluated polarization conditions. Therefore, unlike d_33_ and K_p_, capacitance was less sensitive to changes in domain alignment promoted by temperature and time. This behavior indicates that the polarization process mainly affected the piezoelectric and electromechanical conversion responses, while the dielectric capacitance of the sintered PZT-4 ceramics remained statistically unchanged.

To complement the ANOVA results, Table 8 presents the effect-size parameters calculated for capacitance (C_p_), including eta-squared (η^2^), partial eta-squared (η^2^p), omega-squared (ω^2^), and partial omega-squared (ω^2^p). While ANOVA determines whether the observed differences are statistically significant, effect-size metrics provide additional information regarding the practical magnitude of each factor on the variability of the measured response.

The results indicate that polarization temperature exhibited the largest contribution to the total variability of C_p_, with η^2^ = 0.1383 and η^2^p = 0.1428. Although this contribution can be classified as small to moderate, it was considerably greater than those associated with polarization time and the temperature–time interaction. This observation is consistent with the ANOVA results, where temperature presented the lowest *p*-value among the evaluated factors (*p* = 0.0583), suggesting that temperature was the parameter with the greatest potential influence on capacitance, despite not reaching the adopted significance threshold.

In contrast, polarization time produced extremely low η^2^ and η^2^p values (0.0021 and 0.0025, respectively), indicating a negligible contribution to the observed variability. Similarly, the interaction between temperature and time accounted for only a small fraction of the total variance (η^2^ = 0.0295), reinforcing the conclusion that the combined effects of these parameters did not substantially affect the dielectric response of the material.

The omega-squared parameters provide an even more conservative estimate of effect magnitude. The negative ω^2^ and ω^2^p values obtained for polarization time and interaction are commonly interpreted as null practical effects, indicating that these factors contributed less to the response variability than the experimental error itself. Such results reinforce the statistical evidence that capacitance remained essentially unchanged throughout the investigated polarization conditions.

Overall, the effect-size analysis confirms that capacitance was considerably less sensitive to polarization parameters than the electromechanical properties d_33_ and K_p_. Although temperature exhibited a slightly greater influence than the other factors, its contribution remained insufficient to produce statistically significant changes in C_p_. These findings suggest that the dielectric capacitance of the commercial PZT-4 ceramics remained relatively stable, whereas the primary effects of polarization were manifested through improvements in domain alignment and electromechanical conversion efficiency.

**Figure 14:**
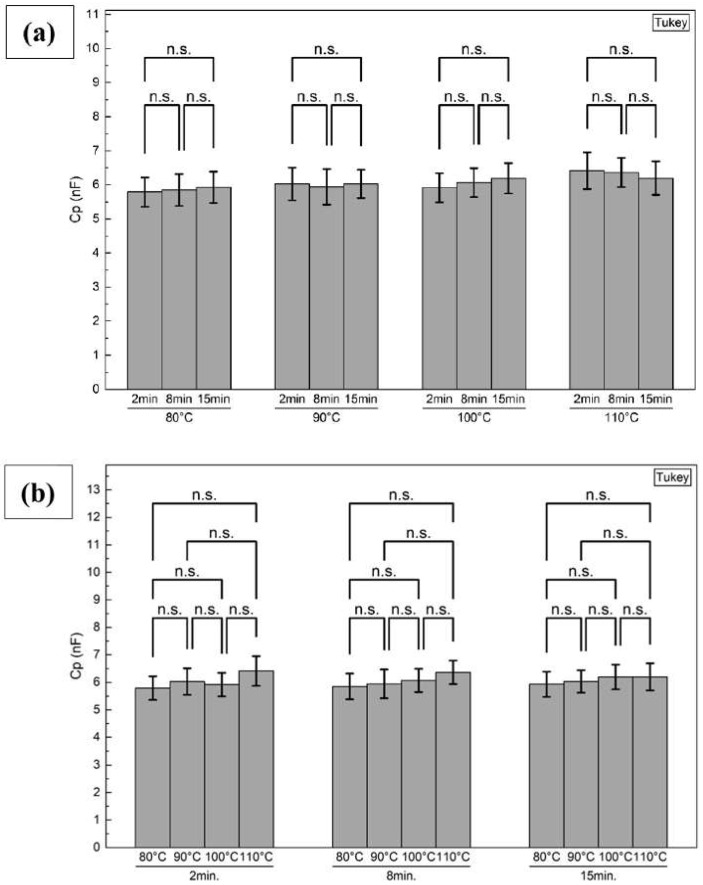
Tukey multiple-comparison test for capacitance (C_p_) of PZT samples. (**a**) Comparison among polarization times within each temperature condition and (**b**) comparison among polarization temperatures within each polarization time. While n.s. indicates non-significant differences.

The multiple-comparison analysis obtained through Tukey’s test is presented in Figure 14. The graphical comparison of capacitance values among the different polarization conditions confirms the statistical tendencies previously observed in the ANOVA and effect-size analyses. In general, no statistically significant differences were identified among the evaluated groups, as indicated by the predominance of non-significant (n.s.) comparisons throughout the dataset.

Figure 14a, which compares polarization times within each temperature condition, reveals that the C_p_ values remained relatively stable as the polarization time increased from 2 to 15 min. Although small fluctuations in the mean values can be observed, the overlap of the standard deviation intervals and the absence of significant Tukey group separations indicate that these variations are within the expected experimental scatter. This behavior suggests that extending the polarization time did not substantially modify the dielectric capacitance of the ceramic, even under conditions that produced noticeable changes in the electromechanical response.

A similar tendency is observed in Figure 14b, where capacitance values are compared among different temperatures for each polarization time. Slight increases in C_p_ can be noted at higher polarization temperatures, particularly for samples polarized at 110 °C. However, these increases were not sufficient to generate statistically significant differences among the groups. This observation is consistent with the ANOVA results, where temperature exhibited the lowest *p*-value among the investigated factors, indicating a tendency toward influence without reaching statistical significance.

From a physical standpoint, the absence of significant differences suggests that the polarization conditions investigated in this work were insufficient to promote substantial modifications in the dielectric storage capability of the material. While higher temperatures and longer polarization times enhanced ferroelectric domain alignment and consequently improved d_33_ and K_p_ values, the overall dielectric response remained comparatively stable. This indicates that the polarization process predominantly affected the orientation and stabilization of ferroelectric domains rather than producing measurable changes in the intrinsic capacitance of the PZT ceramic.

Therefore, the Tukey analysis reinforces the conclusion that capacitance was the least sensitive parameter among the evaluated electromechanical properties. The dielectric behavior remained essentially unchanged throughout the investigated polarization window, demonstrating that the improvements in functional performance observed for d_33_ and K_p_ were achieved without significant alterations in the capacitive characteristics of the material.

Table 9 presents the two-way ANOVA results for the piezoelectric coefficient (d_33_), considering polarization temperature, polarization time, and the interaction between both factors. Unlike the behavior observed for capacitance, the statistical analysis showed that d_33_ was significantly affected by all investigated polarization parameters.

Polarization temperature exhibited the highest F-value (F = 88.94, *p* < 0.0001), indicating that temperature was the most influential factor controlling the piezoelectric response of the PZT ceramics. Polarization time also presented a highly significant effect (F = 72.73, *p* < 0.0001), demonstrating that the duration of the polarization process played an important role in promoting ferroelectric domain alignment. In addition, the temperature–time interaction was statistically significant (F = 7.10, *p* < 0.0001), indicating that the effect of polarization time was dependent on the temperature level applied during the process.

This interaction is particularly relevant because it shows that the influence of time cannot be interpreted independently from temperature. At lower temperatures, longer polarization times are required to progressively increase d_33_, whereas at higher temperatures, domain alignment occurs more rapidly and the piezoelectric response tends to approach saturation. Therefore, the significant interaction confirms that the optimization of d_33_ depends on the combined adjustment of both polarization temperature and time.

From a physical perspective, these results are consistent with the thermally activated behavior of ferroelectric domain-wall motion. Increasing the polarization temperature facilitates domain reorientation by enhancing domain mobility under the applied electric field, while longer polarization times provide additional opportunity for domain switching and stabilization of the polarized state. Consequently, the ANOVA results confirm that the piezoelectric performance of commercial PZT-4 ceramics can be significantly improved through the proper control of polarization temperature and time.

To complement the ANOVA results, Table 10 presents the effect-size analysis for the piezoelectric coefficient (d_33_), including eta-squared (η^2^), partial eta-squared (η^2^p), omega-squared (ω^2^), and partial omega-squared (ω^2^p). These parameters provide quantitative information regarding the magnitude of the effects produced by polarization temperature, polarization time, and their interaction, allowing a more comprehensive interpretation beyond statistical significance alone.

Among the investigated factors, polarization temperature exhibited the largest effect size, with η^2^ = 0.5306 and η^2^p = 0.8475. These values indicate that temperature accounted for approximately 53% of the total variability observed in d_33_ and nearly 85% of the explainable variance when evaluated independently of the other factors. This result confirms that temperature was the dominant variable controlling the piezoelectric response of the investigated PZT-4 ceramics.

Polarization time also exhibited a substantial contribution to the observed variability, with η^2^ = 0.2893 and η^2^p = 0.7519. Although its influence was lower than that of temperature, the results demonstrate that polarization duration remained an important factor in determining the efficiency of ferroelectric domain alignment. The high partial eta-squared value indicates that increasing the polarization time significantly contributed to the development of the piezoelectric response under the investigated experimental conditions.

The interaction between temperature and time presented smaller effect-size values (η^2^ = 0.0847 and η^2^p = 0.4700), indicating that its contribution was secondary compared with the individual effects of temperature and time. Nevertheless, the interaction still explained approximately 8.5% of the total variance and nearly 47% of the variance not associated with the main factors, demonstrating that the combined influence of temperature and time cannot be neglected when optimizing the polarization process.

A similar trend is observed for the omega-squared parameters, which provide a more conservative estimate of effect magnitude. The ω^2^ values confirm the predominance of temperature (ω^2^ = 0.5236), followed by polarization time (ω^2^ = 0.2847), while the interaction term exhibited a comparatively smaller but still relevant contribution (ω^2^ = 0.0726). The close agreement between η^2^ and ω^2^ further reinforces the robustness of the statistical findings.

Overall, the effect-size analysis demonstrates that temperature was the primary factor governing the development of the piezoelectric coefficient, while polarization time exerted a secondary but still substantial influence. The interaction effect, although less pronounced, contributed significantly to the overall behavior of the material. These findings are consistent with the thermally activated nature of ferroelectric domain-wall motion and support the conclusion that elevated temperatures combined with adequate polarization times are essential for maximizing the d_33_ response of commercial PZT-4 ceramics.

**Figure 15:**
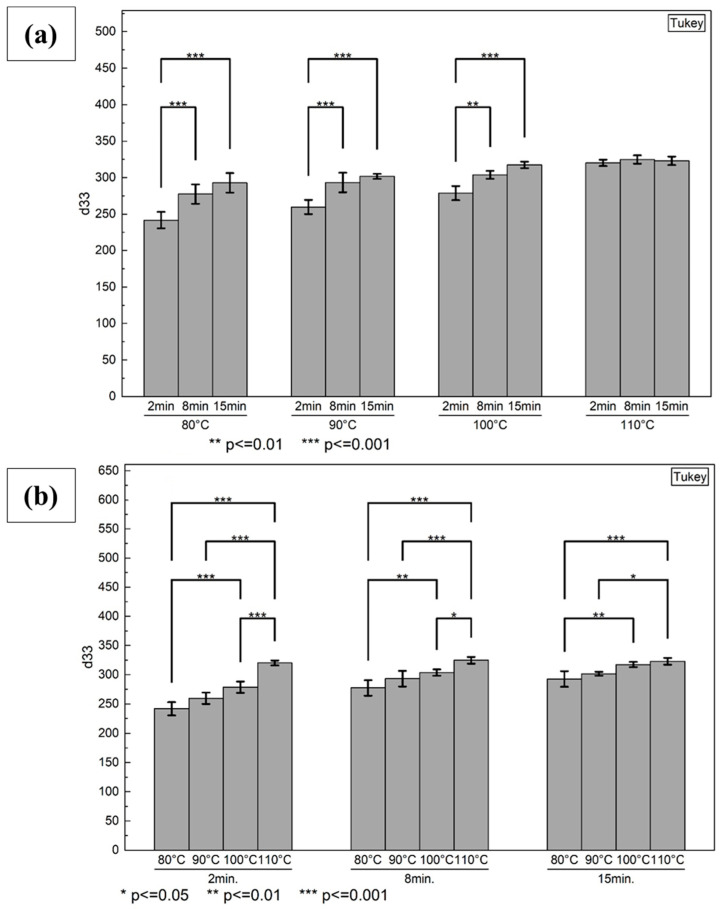
Tukey multiple-comparison test for the piezoelectric coefficient (d_33_) of PZT samples. (**a**) Comparison among polarization times within each temperature condition and (**b**) comparison among polarization temperatures within each polarization time. The symbols *, ** and *** indicate statistical significance at *p* ≤ 0.05, *p* ≤ 0.01 and *p* ≤ 0.001, respectively.

The Tukey multiple-comparison results for the piezoelectric coefficient (d_33_) are presented in Figure 15. The pairwise comparisons provide a detailed evaluation of the differences among polarization conditions and confirm the significant effects of temperature and polarization time identified by the ANOVA.

Figure 15a compares the influence of polarization time within each temperature condition. At 80 °C, significant increases in d_33_ were observed when the polarization time was increased from 2 to 8 min (*p* ≤ 0.001) and from 2 to 15 min (*p* ≤ 0.001), indicating that longer exposure times substantially improved domain alignment under this lower-temperature condition. A similar trend was observed at 90 °C, where the differences between 2 and 8 min and between 2 and 15 min were also highly significant (*p* ≤ 0.001). At 100 °C, the effect of polarization time remained evident, although the difference between 2 and 8 min was slightly less pronounced (*p* ≤ 0.01), while the comparison between 2 and 15 min remained highly significant (*p* ≤ 0.001). In contrast, no statistically significant differences were observed among the polarization times at 110 °C, indicating that the polarization process had already approached saturation even at the shortest treatment time.

The effect of polarization temperature is further illustrated in Figure 15b, where temperatures are compared within each polarization time. For the 2 min condition, significant differences were observed between 80 °C and all higher temperatures, with the strongest significance levels occurring for the comparisons involving 100 and 110 °C (*p* ≤ 0.001). Similar behavior was observed for the 8 min condition, where increasing temperature progressively enhanced the piezoelectric response, with statistically significant differences particularly between the lowest and highest temperature levels. For the 15 min condition, the number of significant differences decreased, indicating that prolonged polarization times partially compensated for the lower thermal activation. Nevertheless, samples polarized at 110 °C still exhibited significantly higher d_33_ values than those polarized at lower temperatures.

An important feature revealed by the Tukey analysis is the progressive reduction in the number of significant differences as both temperature and time increase. This behavior suggests that the material gradually approaches a saturation state in which additional increases in polarization temperature or duration provide only marginal gains in piezoelectric performance. Such a tendency is particularly evident for the samples polarized at 110 °C, where d_33_ values remained statistically similar regardless of polarization time.

From a physical standpoint, these results are consistent with the thermally activated nature of ferroelectric domain-wall motion. At lower temperatures, longer polarization times are required to overcome domain-switching barriers and achieve effective dipole alignment. As the temperature increases, domain mobility becomes progressively greater, accelerating the polarization process and reducing the time necessary to reach a highly aligned ferroelectric state. Consequently, the highest d_33_ values were obtained within the temperature range of 100–110 °C, confirming that this interval represents the most favorable polarization window for maximizing the piezoelectric response of the investigated commercial PZT-4 ceramics.

Table 11 presents the two-way ANOVA results for the electromechanical coupling factor (K_p_), considering polarization temperature, polarization time, and their interaction as independent variables. The statistical analysis revealed that all investigated factors significantly influenced the electromechanical response of the PZT ceramics.

Both polarization temperature and polarization time exhibited highly significant effects on K_p_ (*p* < 0.0001). The F-values obtained for temperature (F = 43.12) and time (F = 64.61) indicate that variations in these parameters produced substantial changes in the electromechanical coupling efficiency of the material. In addition, the interaction between temperature and time was also statistically significant (F = 5.32, *p* = 2.84 × 10^−4^), demonstrating that the effect of one factor depended on the level of the other.

The significance of the interaction term suggests that the influence of polarization time was not constant across all temperatures. Instead, the effectiveness of increasing the polarization duration depended on the thermal condition adopted during polarization. This behavior is consistent with the experimental trends observed in the electromechanical characterization results, where higher temperatures promoted a faster improvement in K_p_ and reduced the additional gains obtained from longer polarization times as the material approached saturation.

The results also indicate that the electromechanical coupling factor is highly sensitive to the polarization process. Since K_p_ is directly associated with the efficiency of energy conversion between electrical and mechanical forms, the significant effects observed for temperature and time demonstrate that both parameters contribute to the establishment of a more favorable ferroelectric domain configuration. As domain alignment becomes more effective, the coupling between electrical excitation and mechanical response is enhanced, resulting in higher K_p_ values.

From a physical perspective, these findings are consistent with the thermally activated behavior of ferroelectric domain-wall motion. Elevated temperatures facilitate domain switching by increasing domain mobility, while longer polarization times provide additional opportunity for dipole alignment and stabilization. Consequently, the ANOVA results confirm that both temperature and time play fundamental roles in determining the electromechanical coupling efficiency of commercial PZT-4 ceramics, highlighting the importance of optimizing both parameters to maximize functional performance.

To further quantify the relative importance of each factor, Table 12 presents the effect-size analysis for the electromechanical coupling factor (K_p_), including eta-squared (η^2^), partial eta-squared (η^2^p), omega-squared (ω^2^), and partial omega-squared (ω^2^p). These parameters provide complementary information to the ANOVA results by evaluating the practical magnitude of the effects associated with polarization temperature, polarization time, and their interaction.

The results indicate that polarization temperature (η^2^ = 0.3822) and polarization time (η^2^ = 0.3817) contributed almost equally to the total variability observed in K_p_. This behavior differs from that observed for the piezoelectric coefficient d_33_, where temperature was clearly the dominant factor. For K_p_, both variables exhibited comparable influence, demonstrating that the electromechanical coupling efficiency depends not only on the thermal activation of domain-wall motion but also on the duration of the polarization process.

The partial eta-squared values further reinforce this interpretation. Both temperature (η^2^p = 0.7294) and polarization time (η^2^p = 0.7291) exhibited very large effect sizes, indicating that each factor independently explained approximately 73% of the variance not associated with the remaining sources of variation. These values confirm that both parameters played fundamental roles in determining the electromechanical coupling performance of the investigated PZT ceramics.

The interaction between temperature and time exhibited smaller effect-size values (η^2^ = 0.0943 and η^2^p = 0.3994), indicating a secondary but still meaningful contribution to the overall variability of K_p_. Although less influential than the main factors, the interaction effect demonstrates that the response of the material cannot be fully explained by considering temperature and time independently. Instead, the effectiveness of increasing one parameter depends partially on the level of the other, corroborating the interaction identified in the ANOVA analysis.

A similar trend is observed for the omega-squared coefficients, which provide a more conservative estimate of effect magnitude. The values obtained for temperature (ω^2^ = 0.3722) and time (ω^2^ = 0.3747) remained remarkably similar, confirming the balanced influence of both factors on the electromechanical coupling factor. Meanwhile, the interaction term exhibited a smaller contribution (ω^2^ = 0.0764), although still sufficient to indicate a measurable combined effect.

Overall, the effect-size analysis demonstrates that polarization temperature and polarization time contributed almost equally to the development of the electromechanical coupling factor. These findings suggest that maximizing K_p_ requires a simultaneous optimization of both parameters, since neither factor alone is sufficient to fully explain the observed improvements in electromechanical performance. This behavior is consistent with the nature of K_p_, which reflects the efficiency of energy conversion and therefore depends on both the degree of domain alignment achieved and the stability of the polarized ferroelectric state.

**Figure 16:**
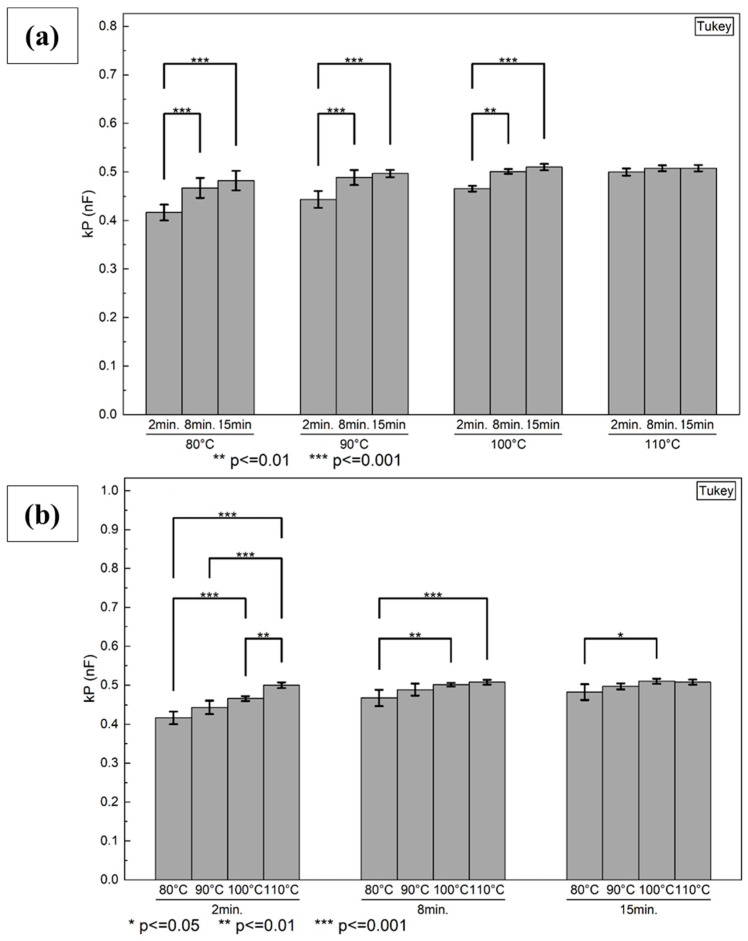
Tukey multiple-comparison test for the electromechanical coupling factor (K_p_) of PZT samples. (**a**) Comparison among polarization times within each temperature condition and (**b**) comparison among polarization temperatures within each polarization time. The symbols *, ** and *** indicate statistical significance at *p* ≤ 0.05, *p* ≤ 0.01 and *p* ≤ 0.001, respectively.

The Tukey multiple-comparison analysis for the electromechanical coupling factor (K_p_) is presented in Figure 16. The results provide a detailed assessment of the differences among polarization conditions and confirm the significant effects of temperature and polarization time identified by the ANOVA.

Figure 16a compares the influence of polarization time within each temperature condition. At 80 °C, statistically significant increases in K_p_ were observed when the polarization time increased from 2 to 8 min (*p* ≤ 0.001) and from 2 to 15 min (*p* ≤ 0.001), demonstrating that prolonged polarization promoted a substantial improvement in electromechanical coupling under limited thermal activation. A similar behavior was observed at 90 °C, where the comparisons between 2 and 8 min and between 2 and 15 min remained highly significant (*p* ≤ 0.001). At 100 °C, significant differences were also detected, although the effect became less pronounced, with the comparison between 2 and 8 min showing a significance level of *p* ≤ 0.01 and the comparison between 2 and 15 min remaining highly significant (*p* ≤ 0.001). In contrast, no statistically significant differences were observed among the polarization times at 110 °C, indicating that the electromechanical coupling factor had already approached its maximum attainable values under the shortest polarization treatment.

The influence of polarization temperature is illustrated in Figure 16b. For the 2 min condition, K_p_ increased significantly as the temperature increased, with highly significant differences observed between 80 °C and the higher temperature conditions. The strongest statistical differences occurred between 80 °C and 110 °C (*p* ≤ 0.001), confirming the substantial contribution of thermal activation to the development of electromechanical coupling. For the 8 min condition, the same trend was maintained, with significant differences observed mainly between the lowest and highest temperatures. However, for the 15 min condition, the number of statistically significant comparisons was considerably reduced, indicating that extended polarization times partially compensated for the lower temperatures and promoted convergence of the K_p_ values.

An important feature revealed by the Tukey analysis is the progressive reduction in significant differences as both temperature and polarization time increase. This behavior indicates that the material tends toward an electromechanical saturation state, where additional increases in temperature or polarization duration result in progressively smaller gains in K_p_. Such behavior is particularly evident for the samples polarized at 100 and 110 °C, whose K_p_ values remained close to 0.50 regardless of further increases in polarization time.

From a physical standpoint, these findings are consistent with the mechanisms governing ferroelectric domain alignment. Higher temperatures facilitate domain-wall motion and reduce the energy barriers associated with dipole reorientation, while longer polarization times allow a greater fraction of domains to become aligned and stabilized under the applied electric field. As a consequence, the efficiency of electromechanical energy conversion increases progressively until a near-saturated polarization state is reached. The results therefore confirm that the most favorable polarization conditions for maximizing K_p_ are concentrated within the temperature range of 100–110 °C and polarization times between 8 and 15 min, in agreement with the trends previously observed for the piezoelectric coefficient d_33_.

## 4. Conclusions

The present study demonstrated that the functional performance of commercial Sparkler PZT-4 (Navy Type I) ceramics is strongly governed by the interrelation between powder morphology, densification behavior, crystallographic stability, and polarization parameters. SEM analyses revealed a heterogeneous and polydisperse powder composed of hierarchical agglomerates formed by micrometric faceted primary particles. Such agglomeration may influence particle packing during compaction, potentially generating local densification heterogeneities and residual porosity that can affect electric-field homogeneity during polarization. Despite this initial morphology, the adopted thermal treatment at 1230 °C promoted satisfactory consolidation, resulting in ceramics with high apparent density (7.68 g/cm^3^), low water absorption (0.12%), low apparent porosity (0.91%), and a relative density of approximately 96%, evidencing effective densification and structural integrity. The high densification achieved after sintering minimized the detrimental effects commonly associated with agglomerated powders, contributing to efficient domain alignment and the elevated electromechanical performance observed in the polarized ceramics.

X-ray diffraction confirmed the predominance of the ferroelectric perovskite Pb(Zr,Ti)O_3_ phase with tetragonal symmetry (P4mm), consistent with compositions close to the morphotropic phase boundary, which is favorable for enhanced electromechanical properties. Rietveld refinement indicated high crystallinity and only minor traces of secondary ZrO_2_, suggesting that the sintering route was efficient in preserving the functional crystalline structure responsible for the piezoelectric response.

The electromechanical characterization showed that polarization temperature and time significantly affected the material response, whereas capacitance remained comparatively stable. The highest piezoelectric coefficient was obtained under 110 °C for 15 min and 3.0 kV/mm, reaching d_33_ = 325.8 pC/N, while the highest coupling factors were observed between 100 and 110 °C, with K_p_ values up to 0.509. These results demonstrate that the optimum condition depends on the targeted property: maximum d_33_ was obtained at a high temperature and longer polarization time, whereas higher temperatures provided superior electromechanical coupling stability.

The Avrami kinetic approach proved suitable for describing the domain alignment process during polarization. The phenomenological Avrami fitting adequately described the apparent evolution of polarization efficiency under different processing conditions. Higher polarization temperatures produced fitting curves that approached saturation at shorter polarization times, indicating a more rapid evolution of the measured electromechanical response. The fitting parameters obtained from the model were used exclusively for comparative purposes and were not interpreted in terms of classical nucleation-and-growth mechanisms. At 110 °C, transformed fractions close to unity were achieved even at short times, confirming that temperature is the dominant variable controlling domain reorientation efficiency.

Statistical analyses by ANOVA and Tukey tests validated the significant influence of polarization conditions on d_33_ and K_p_, while C_p_ exhibited no statistically relevant differences. The combined experimental results indicate that polarization temperatures between 100 and 110 °C and polarization times between 8 and 15 min under a fixed electric field of 3.0 kV/mm produced the highest electromechanical performance. Within this polarization window, d_33_ values above 300 pC/N and K_p_ values above 0.50 were consistently obtained. Therefore, the present study identifies favorable polarization conditions within the investigated range of commercial PZT-4 ceramics under the investigated polarization regime. From an application standpoint, the investigated material showed excellent potential for sensors, actuators, ultrasonic transducers, and high-power piezoelectric devices requiring elevated electromechanical reliability and stable functional performance.

Although the present study provides a consistent evaluation of the influence of polarization parameters on the electromechanical behavior of commercial PZT-4 ceramics, additional functional analyses such as dielectric loss (tanδ), temperature-dependent dielectric spectroscopy, impedance spectroscopy, dielectric breakdown strength, and long-term thermal aging stability would further contribute to a more comprehensive understanding of the material performance under service conditions. These characterizations are considered important perspectives for future investigations.

## Figures and Tables

**Table 1:** Experimental design of polarization parameters (time, temperature, and electric field) for sintered PZT samples.

Samples Groups	Time (min)	Temperature (°C)	Electric Field (kV/mm)
SS-2/80/3	2.0	80	3.0
SS-8/80/3	8.0	80	3.0
SS-15/80/3	15.0	80	3.0
SS-2/90/3	2.0	90	3.0
SS-8/90/3	8.0	90	3.0
SS-15/90/3	15.0	90	3.0
SS-2/100/3	2.0	100	3.0
SS-8/100/3	8.0	100	3.0
SS-15/100/3	15.0	100	3.0
SS-2/110/3	2.0	110	3.0
SS-8/110/3	8.0	110	3.0
SS-15/110/3	15.0	110	3.0

**Table 2:** Elemental composition of PZT-Sparkler determined by EDS.

Element	C. Norm. [wt. %]	Error [%]
Lead	64.72	17.52
Zirconium	10.73	2.22
Titanium	7.10	1.70
Oxygen	10.85	14.12
Carbon	6.59	10.49

**Table 3:** Physical properties observed for sintered PZT samples.

Samples	SM (g)	WW (g)	DW (g)	WA (%)	AP (%)	D_a_ (g/cm^3^)	D_r_ (%)
SS	16.15 ± 0.24	18.55 ± 0.28	18.54 ± 0.27	0.12 ± 0.08	0.91 ± 0.66	7.68 ± 0.06	96.02 ± 0.87

**Table 4:** PZT pattern 01-070-4060 parameters.

Reference Code: 01-070-4060 Compound Name: Lead Zirconium Titanium Oxide					
Crystallographic Parameters		Peak List			
		hkl	d(Å)	2θ	I (%)
Empirical Formula	O_3_PbTi_0.48_Zr_0.52_	001	4.110	21.605	17.2
Chemical Formula	Pb(Zr_0.52_Ti_0.48_)O_3_	100	4.055	21.901	22.9
Crystal System	Tetragonal	101	2.886	30.955	100.0
Space Group	P4mm	110	2.867	31.168	54.7
Space Group Nº	99	111	2.351	38.242	25.0
a (Å)	4.0550	002	2.055	44.029	10.4
b (Å)	4.0550	200	2.027	44.658	24.9
c (Å)	4.1100	102	1.833	49.698	8.6
α (°)	90	201	1.818	50.129	5.9
β (°)	90	112	1.670	54.925	14.3
γ (°)	90	211	1.659	55.327	28.9
Calculated Density (g/cm^3^)	8.00	220	1.433	64.999	5.9
Volume of Cell (10^6^ pm)	67.58	221	1.353	69.367	1.9
		103	1.297	72.810	3.9
		310	1.282	73.842	5.6

**Table 5:** Summary of the electromechanical properties calculated for each group of samples.

Samples Groups	C_p_ (nF)	d_33_ (pC/N)	K_p_
SS-2/80/3	5.788 ± 0.430	241.7 ± 11.1	0.416 ± 0.016
SS-8/80/3	5.850 ± 0.467	277.4 ± 12.8	0.467 ± 0.020
SS-15/80/3	5.928 ± 0.460	292.7 ± 14.6	0.482 ± 0.020
SS-2/90/3	6.028 ± 0.482	259.7 ± 10.2	0.443 ± 0.017
SS-8/90/3	5.942 ± 0.522	293.3 ± 13.6	0.488 ± 0.015
SS-15/90/3	6.030 ± 0.409	301.7 ± 3.4	0.496 ± 0.007
SS-2/100/3	5.916 ± 0.427	278.7 ± 10.9	0.465 ± 0.005
SS-8/100/3	6.068 ± 0.425	303.8 ± 5.5	0.501 ± 0.004
SS-15/100/3	6.191 ± 0.450	317.4 ± 5.3	0.509 ± 0.006
SS-2/110/3	6.411 ± 0.538	320.2 ± 5.2	0.499 ± 0.007
SS-8/110/3	6.372 ± 0.453	324.7 ± 4.2	0.507 ± 0.006
SS-15/110/3	6.196 ± 0.492	325.8 ± 3.9	0.507 ± 0.006

**Table 6:** Avrami kinetic parameters for domain alignment in PZT samples under different polarization time and temperature conditions.

Samples Groups	d_33_	Y(t)	*k*	*n*
SS-2/80/3	241.7	0.73	1.12	0.23906
SS-8/80/3	277.4	0.84		
SS-15/80/3	292.7	0.89		
SS-2/90/3	259.7	0.79	1.31	0.25006
SS-8/90/3	293.3	0.89		
SS-15/90/3	301.7	0.91		
SS-2/100/3	278.7	0.84	1.56	0.23141
SS-8/100/3	303.8	0.92		
SS-15/100/3	317.4	0.96		
SS-2/110/3	320.2	0.97	3.24	0.11619
SS-8/110/3	324.7	0.98		
SS-15/110/3	325.8	0.99		

**Table 7:** Two-way ANOVA results for capacitance (C_p_) considering the effects of polarization temperature, polarization time, and their interaction.

	DF	Sum of Squares	Mean Square	F Value	*p* Value
**Temperature**	3	1.71248	0.57083	2.66582	0.05827
**Time**	2	0.0262	0.0131	0.06118	0.94073
**Interaction**	6	0.36528	0.06088	0.28431	0.9416
**Model**	11	2.10396	0.19127	0.89325	0.55329
**Error**	48	10.27817	0.21413		
**Corrected Total**	59	12.38213			

**Table 8:** Effect size analysis for capacitance (C_p_) based on eta-squared (η^2^), partial eta-squared (η^2^p), omega-squared (ω^2^), and partial omega-squared (ω^2^p) values.

	a	b	c	d
**Temperature**	0.1383	0.14282	0.08495	0.07689
**Time**	0.00212	0.00254	−0.03192	−0.0323
**Interaction**	0.0295	0.03432	−0.073	−0.07709

^a^ Eta-Squared; ^b^ Partial Eta-Squared; ^c^ Omega-Squared; ^d^ Partial Omega-Squared.

**Table 9:** Two-way ANOVA results for the piezoelectric coefficient (d_33_) considering the effects of polarization temperature, polarization time, and their interaction on the electromechanical response of commercial PZT-4 ceramics.

	DF	Sum of Squares	Mean Square	F Value	*p* Value
**Temperature**	3	22,271.04583	7423.68194	88.93744	<0.0001
**Time**	2	12,141.175	6070.5875	72.72705	<0.0001
**Interaction**	6	3553.39167	592.23194	7.09508	<0.0001
**Model**	11	37,965.6125	3451.41932	41.34881	<0.0001
**Error**	48	4006.6	83.47083		
**Corrected Total**	59	41,972.2125			

**Table 10:** Effect size analysis for the piezoelectric coefficient (d_33_) considering the contributions of polarization temperature, polarization time, and their interaction based on eta-squared (η^2^), partial eta-squared (η^2^p), omega-squared (ω^2^), and partial omega-squared (ω^2^p).

	a	b	c	d
**Temperature**	0.53061	0.84753	0.52361	0.81471
**Time**	0.28927	0.75188	0.2 8472	0.70509
**Interaction**	0.08466	0.47003	0.07258	0.37869

^a^ Eta-Squared; ^b^ Partial Eta-Squared; ^c^ Omega-Squared; ^d^ Partial Omega-Squared.

**Table 11:** Two-way ANOVA results for the electromechanical coupling factor (K_p_) considering the effects of polarization temperature, polarization time, and their interaction.

	DF	Sum of Squares	Mean Square	F Value	*p* Value
**Temperature**	3	0.02074	0.05426	43.11903	<0.0001
**Time**	2	0.02071	0.00691	64.60507	<0.0001
**Interaction**	6	0.00512	8.52782 × 10^−4^	5.31997	2.83663 × 10^−4^
**Model**	11	0.04656	8.52782 × 10^−4^	26.40791	<0.0001
**Error**	48	0.00769	1.60298 × 10^−4^		
**Corrected Total**	59	0.05426			

**Table 12:** Two-way ANOVA results for the electromechanical coupling factor (K_p_) as a function of polarization temperature, polarization time, and their interaction.

	a	b	c	d
**Temperature**	0.38216	0.72936	0.3722	0.67804
**Time**	0.38173	0.72914	0.37471	0.6795
**Interaction**	0.0943	0.3994	0.07635	0.30167

^a^ Eta-Squared; ^b^ Partial Eta-Squared; ^c^ Omega-Squared; ^d^ Partial Omega-Squared.

## Data Availability

The original contributions presented in the study are included in the article; further inquiries can be directed to the corresponding author.
